# Flood hazard assessment and zonal prioritization through an LR-bipolar triangular fuzzy hybrid decision-making approach

**DOI:** 10.3389/frai.2026.1817216

**Published:** 2026-05-01

**Authors:** Ajeesh Puthusserry Paulose, Felix Augustin

**Affiliations:** Department of Mathematics, School of Advanced Sciences, Vellore Institute of Technology, Chennai, Tamil Nadu, India

**Keywords:** combinative distance-based assessment, flood-prone zones, LR-bipolar triangular fuzzy number, preference selection index, simple additive weighting

## Abstract

**Introduction:**

Flood risk assessment has become increasingly important in regions vulnerable to climate-induced disasters. This study addresses the need for a robust decision-support framework by proposing a hybrid multi-criteria decision-making (MCDM) model to prioritize flood-prone zones in the Ernakulam district of Kerala, aligning with sustainable development goals focused on climate resilience.

**Methods:**

The proposed approach employs LR-bipolar triangular fuzzy numbers (LRBTFNs) to effectively capture uncertainty in decision-making. It integrates three well-established MCDM techniques, Preference Selection Index (PSI), Simple Additive Weighting (SAW), and Combinative Distance-based Assessment (CODAS), to ensure a balanced and comprehensive ranking process. The methodology incorporates normalization, aggregation, and defuzzification steps to compute performance scores. Additionally, unsupervised learning techniques, namely K-means clustering and Principal Component Analysis (PCA), are utilized to validate vulnerability patterns and group regional profiles.

**Results and discussion:**

The results reveal that Kochi, Vypen, and Paravoor are the most vulnerable flood-prone zones, while Kothamangalam is identified as the least susceptible area. The integration of multiple MCDM methods enhances the robustness and reliability of the ranking outcomes, and clustering and PCA analyses further confirm consistent vulnerability trends across regions. The findings provide valuable insights for policymakers and local authorities to implement targeted risk mitigation and planning strategies. Moreover, the study supports Sustainable Development Goal 13 (Climate Action) by promoting resilience and preparedness against climate-induced flood hazards.

## 1 Introduction

Floods are among the most frequent and devastating natural disasters globally, often triggered by prolonged or intense rainfall. Historically, flooding has remained a predominant natural hazard, with its impact exacerbated by unplanned urbanization, deforestation, ill-conceived land-use changes, and unscientific modifications to natural drainage systems (Rogger et al., 2017). Factors such as climate change, rapid urban expansion, and reduced vegetation cover have significantly contributed to the increasing frequency and severity of floods. Short-duration but high-intensity rainfall events further aggravate flood risks, manifesting in various forms such as coastal, urban, and flash floods (Zhang et al., 2022).

Over the past three decades, flood incidents have intensified, causing severe disruptions to infrastructure, transportation, utilities, and agricultural sectors. These events not only result in economic losses but also lead to interruptions in public services, outbreaks of waterborne diseases, and widespread psychological distress (Wu et al., 2024). The 2020 global natural disaster assessment report highlighted that floods have been the most dominant natural hazard, responsible for a substantial number of fatalities, property destruction, and economic damages worldwide. In particular, when compared globally, the most flood-affected countries between 1900 and 2022 are in Asia, according to the EM-DAT statistics (Nakasu and Amrapala, 2023). Asia continues to experience the highest number of flood events globally, with increasing mortality rates attributed to climate-induced phenomena such as global warming, glacial and polar ice melt, and rising sea levels (Wang et al., 2024). These alarming trends necessitate urgent and comprehensive flood risk assessment and management frameworks.

India, like many other disaster-prone regions, frequently faces severe flooding events. Its diverse topography and climatic conditions render approximately 85% of its landmass vulnerable to natural hazards. Over the last few decades, India has experienced numerous flood events, resulting in significant human and economic losses (Mohanty et al., 2020). Over the span of 64 years, from 1953 to 2017, India experienced 107,535 fatalities as a result of heavy rainfall and flooding, according to a report by the central water commission (CWC), Government of India. During this period, damage to public infrastructure, housing, and agriculture was estimated at approximately USD 53.43 billion (INR 378,247.047 crores) (Mangukiya et al., 2022).

Particularly, the western coastal state of Kerala is highly susceptible to floods due to its unique geography, being flanked by the Western Ghats on one side and the Arabian Sea on the other. Kerala, located in southwestern India, the state has a humid tropical climate and receives its first monsoon rains in late May or early June, averaging 3,107 mm annually. Rainfall varies from 1,250 mm in lowlands to 5,000 mm in highlands due to orographic effects, with about 120–140 rainy days per year. Its climate is shaped by the Southwest monsoon (June–August), which brings around 65% of annual rainfall, and the Northeast monsoon (September–December). The Southwest monsoon first strikes Kerala, making it the initial recipient of monsoon rains in India. With 44 monsoon-fed rivers and steep terrain, Kerala is prone to floods. Riverine flooding is common during the monsoon, while flash floods occur in the Western Ghats and urban flooding affects cities like Kochi and Thiruvananthapuram, exacerbated by intense rain and poor drainage. Major floods in 2018, 2019, 2020, and 2024 highlight the growing severity and impact on lives and infrastructure (Singh et al., 2025).

To minimize flood-induced damage, it is essential to implement effective modeling, forecasting, and mitigation strategies. A key component in such efforts is the development of flood susceptibility maps, which depend on efficiently identifying vulnerable regions (Saravanan et al., 2023). Given the inherently uncertain and multi-dimensional nature of flood risk factors, fuzzy logic offers a powerful means of modeling ambiguity and subjectivity in decision-making processes. Since flood risk assessment involves evaluating multiple and often conflicting criteria, the multi-criteria decision-making (MCDM) techniques are especially suitable to model the problem of identifying flood-prone zones.

Fuzzy MCDM models are particularly valuable for handling vague, imprecise, and incomplete information, which is often the case in expert-driven evaluations. Fuzzy logic, a generalization of classical logic derived from fuzzy set theory, accommodates approximate reasoning and is well-suited for uncertain environments. Furthermore, bipolar fuzzy numbers extend the classical fuzzy numbers, incorporating positive and negative membership functions, offering a more comprehensive representation of uncertainty in decision analysis. Recently, researchers have focused on developing bipolar fuzzy decision-making models (Nithyanandham and Augustin, 2023; Natarajan et al., 2023; Nithyanandham et al., 2023a; Jana et al., 2024b,a; Zararsız, 2024; Natarajan and Augustin, 2024; Natarajan et al., 2024; Nithyanandham et al., 2025a; Nithyanandham and Augustin, 2025; Nithyanandham et al., 2025b) for effective decision analysis. The LR-bipolar triangular fuzzy number offers more flexibility in representing uncertainty for handling real-world problems (Chakraborty and Saha, 2022; Sampathkumar et al., 2025).

Among the various MCDM approaches, the combinative distance-based assessment (CODAS) method (Ghorabaee et al., 2016) is notable for utilizing both Euclidean and Taxicab distances in evaluating alternatives. Meanwhile, the preference selection index (PSI) method provides an efficient mechanism to compute weights of the criteria objectively. Additionally, the simple additive weighting (SAW) method is widely used for aggregating weighted criteria to derive composite scores.

In light of these considerations, this study proposes a hybrid PSI–SAW–CODAS framework under the LR-bipolar triangular fuzzy number (LRBTFN) environment to identify and rank of flood-susceptible zones in the Ernakulam district of Kerala. The proposed model considers 14 flood-prone areas as alternatives and evaluates them based on 10 critical criteria: altitude, historical flood records, rainfall, soil type, land use and urbanization, ground slope, existing flood control measures, proximity to water bodies, population density, and green cover. The subsequent section presents a review of relevant literature focusing on MCDM-based flood risk assessment techniques.

## 2 Literature review

Flood risk assessment (FRA) has become a crucial aspect of environmental and urban planning due to increasing climatic uncertainties. Floods are among the most frequent natural disasters worldwide, affecting diverse regions and socio-economic conditions. Researchers have increasingly used MCDM techniques (Lyu et al., 2023) to map and manage flood vulnerabilities in various regions of the world. In the decision-making process, MCDM models allow decision-makers to assess, rank, prioritize, and identify a set of alternatives with respect to multiple and conflicting criteria.

The analytic hierarchy process (AHP) and fuzzy AHP (FAHP) have been widely adopted in early flood-prone zone studies, providing structured pairwise comparisons of criteria. To address spatial flood vulnerability issues, De Brito et al. (2018) applied AHP and analytic network process models. They also estimated the applicability of these methods to be about 75% and 80% in FRA due to their user-friendly nature. Vignesh et al. (2021) applied the AHP model with remote sensing and geographic information system (GIS) techniques to identify flood-prone zones (FPZs) in Kanyakumari district, India. Wu et al. (2022) employed AHP with the entropy weighting technique for short- and long-term FRA in China and noted that the northeastern regions were more vulnerable with respect to key criteria. Zheng et al. (2022) integrated Grey theory and DEMATEL into AHP, simplified as G-DEMATEL-AHP, to identify the most critical inundation risk areas. They also conducted a comparative study of G-DEMATEL-AHP and FAHP and revealed that the former is more effective. Abdrabo et al. (2023) developed a composite indicator using principal component analysis (PCA) to identify the flood vulnerability index (FVI) in Alexandria city, Egypt, and compared it with AHP, concluding that El-Gomrok exhibited the highest FVI in both methods.

Although many authors apply AHP for assessing FRA and FVI, it struggles with inconsistencies in expert opinions, particularly in complex or uncertain environments. Thus, the FAHP model has been considered to overcome these issues. Lyu et al. (2020) incorporated AHP and FAHP with GIS for inundation risk assessment in the Shenzhen metro system, China. Vilasan and Kapse (2022) evaluated the prediction capability of AHP and FAHP in assessing flood vulnerability in the Ernakulam district, Kerala. They concluded that 19% of the district is highly vulnerable due to factors such as low slope gradient, reduced stream capacity, higher soil moisture content, low infiltration capacity, and anthropogenic interventions. Senan et al. (2023) assessed and compared AHP and F-AHP models for identifying flood-vulnerable zones in Kottayam district, Kerala, and concluded that AHP performed better in that context. Guan et al. (2024) employed FAHP with a random forest algorithm for FRA in the Zhengzhou metro system, China. Recently, Taoukidou et al. (2025) conducted a comparative FRA using AHP, FAHP, and frequency ratio models in Greece. Sood et al. (2025) utilized FAHP based on GIS to identify FPZs in Chennai, India, considering historical flood records.

Researchers have also applied a variety of MCDM models to flood-related problems. Nithyanandham et al. (2023b) developed a bipolar intuitionistic fuzzy digraph-based decision-making system to assess FPZs in Chennai, India. Mohammadifar et al. (2023) employed deep learning models along with MCDM techniques such as CODAS, EDAS, and MOOSRA to analyze flood risk maps in Southern Iran.

The literature on flood susceptibility and hazard assessment has recently expanded through various approaches, including both hybrid MCDM frameworks and AI-driven models. In terms of AI-based approaches, recent studies demonstrate the effectiveness of machine learning and deep learning in improving prediction accuracy, real-time flood monitoring, and decision-making under uncertainty, particularly through models such as Random Forest, XGBoost, ANN, GIS, and GeoAI-enhanced algorithms (Bari et al., 2023; Wang et al., 2025; Mabrouk et al., 2025; Waleed and Sajjad, 2024; Adeyemi and Komolafe, 2025). These works highlight the ability of AI to process multi-source data (e.g., satellite, hydrological, climatic) and provide scalable, explainable, and high-accuracy flood susceptibility maps. Concurrently, hybrid MCDM models have gained prominence by integrating decision-making techniques with machine learning or GIS frameworks to enhance robustness and interpretability. Notable contributions include AHP-based machine learning hybrids, fuzzy MCDM integrations such as FAHP–FTOPSIS, collaborative GIS-based MCDA frameworks, AHP-VIKOR hybrid models, and advanced fuzzy-AHP–ML combinations for flood mapping (Singha et al., 2025a; Rizalihadi et al., 2026; Radmehr, 2025; Barman et al., 2024; Singha et al., 2025b). These hybrid approaches effectively combine expert judgment with data-driven learning, improving both the reliability and practical applicability of flood risk assessment models. The widespread adoption of AHP in these studies is attributed to its ability to systematically derive criteria weights from expert judgments while ensuring consistency and transparency, particularly in complex flood susceptibility problems involving both qualitative and quantitative factors. Furthermore, AHP serves as a flexible foundation that can be seamlessly integrated with fuzzy logic, GIS, and machine learning techniques, thereby enhancing the robustness and interpretability of hybrid flood risk assessment models.

### 2.1 Research gaps

Despite the extensive use of classical and fuzzy MCDM approaches in flood risk assessment, several critical research gaps remain as follows:

(i) Many existing studies rely on single-method frameworks (e.g., AHP or FAHP), which may suffer from subjectivity in weighting or lack robustness in handling conflicting criteria.
(ii) Although some studies employ hybrid models, they often do not systematically integrate objective weighting mechanisms with robust ranking strategies.
(iii) Most of the existing fuzzy approaches focus on single-sided uncertainty representation, limiting their ability to simultaneously capture positive and negative assessments inherent in real-world flood risk evaluation.

In this context, the integration of PSI, SAW, and CODAS under an LR-bipolar triangular fuzzy environment offers a significant advantage. The PSI method provides objective and data-driven weights based on dispersion characteristics, thereby reducing reliance on subjective judgments. The SAW method ensures a simple yet effective aggregation mechanism that preserves computational efficiency. The CODAS method, being distance-based, enhances discrimination power by considering both Euclidean and Hamming distances from the negative ideal solution. Furthermore, the adoption of LRBTFNs enables simultaneous modeling of positive and negative uncertainties with greater flexibility compared to traditional fuzzy or intuitionistic fuzzy frameworks. This integrated framework thus addresses the limitations of existing models by improving robustness, discrimination ability, and realism in representing uncertainty.

### 2.2 Motivations of the research

In recent years, hybrid fuzzy models have gained popularity in handling complex decision-making scenarios. Despite their advantages, many of the aforementioned models that assess flood-related issues focus primarily on flood risk criteria or identifying zones. However, considering both zones and flood risk criteria in such problems greatly increases the reliability and efficiency of the study. Moreover, the consideration of the importance of different flood risk criteria is crucial in FPZ analysis. Compared to subjective weights, objective weighting provides more meaningful insights in such assessments. Although several fuzzy MCDM models exist, distance-based approaches possess strong theoretical foundations. Compared to classical fuzzy approaches, the bipolar fuzzy approach significantly facilitates the investigation of problems from both positive and negative perspectives. LR fuzzy numbers provide more flexibility in representing uncertainty in real-world problems. While individual methods have their respective strengths, hybridizing multiple techniques into a unified decision-making framework offers a more effective solution.

As per many reports, India faces multiple natural hazards due to floods. In India, Kerala is one of the most flood-affected states due to its geographical characteristics. Although some studies have addressed FVI and FRA in Kerala, the identification of FPZs by considering multiple and conflicting criteria has not been extensively explored. Moreover, the increasing frequency and severity of flood events underscore the urgent need for data-driven, sustainable, and resilient urban management strategies. In alignment with the United Nations Sustainable Development Goals (SDGs), particularly SDG 11 (Sustainable Cities and Communities) and SDG 13 (Climate Action), this study contributes to global efforts aimed at enhancing disaster resilience and adaptive planning in vulnerable regions. The proposed framework supports evidence-based decision-making for flood risk mitigation and sustainable land-use management, thereby facilitating the development of safer and more climate-resilient communities.

From these important issues and key motivations, the present study employs several novel contributions as described below.

(i) The notion of LRBTFN is defined to flexibly represent uncertainty.
(ii) The arithmetic operations on LRBTFNs are defined to support the theoretical framework.
(iii) A hybrid decision-making system is designed using PSI, SAW, and CODAS approaches under a LR-bipolar triangular fuzzy (LRBTF) context.
(iv) The Euclidean and Hamming distance measures are extended under the BLRTF environment.
(v) An application is presented to identify flood-prone zones in Ernakulam district, Kerala.
(vi) A comparative analysis is performed to demonstrate the effectiveness of the proposed model.
(vii) Finally, K-means clustering and PCA methods are applied for further validation of the results.

### 2.3 Novelty of the proposed work

The novelty of this study lies not merely in combining existing methods, but in extending them within a unified LRBTF framework. Specifically, the study introduces:

- A structured representation of LRBTFNs,
- Arithmetic operations under this environment,
- A score function for the LRBTFNs.
- Extension of distance measures for LRBTFNs,
- Integration of PSI-based objective weighting within this context.

These contributions collectively provide a more flexible and comprehensive decision-making framework compared with existing approaches.

Organization of the manuscript: In Section 1, the introduction of the work is provided. In Section 2, the literature review is given with the motivation and contribution of the research. In Section 3, the preliminary definitions of the work are discussed. In Section 4, the proposed decision-making model is given. In Section 5, the case study is discussed with the implementation. In Section 6, the results are discussed with several insights. Finally, the conclusion and future scope of the work are discussed in Section 7.

## 3 Preliminaries

In this part, several fundamental definitions are provided to support the development of the proposed decision-making model.

**Definition 3.1.** *Let X be a universe of discourse. Then, a bipolar fuzzy set* $\tilde{B}$ *is defined as*


$$\begin{array}{l} \tilde{B}=\left\{\left(x,\mu_{\tilde{B}}^{+}\left(x\right),\mu_{\tilde{B}}^{-}\left(x\right)\right)|x\in X\right\}, \end{array}$$

*where* $\mu_{\tilde{B}}^{+}\left(x\right):X\to \left[0,1\right]$ and $\mu_{\tilde{B}}^{-}\left(x\right):X\to \left[-1,0\right]$ *represent the positive and negative membership degrees of the bipolar fuzzy set* $\tilde{B}$, *respectively*.

Conventional TFNs represent uncertainty using a single membership function, which may not adequately capture situations where both favorable and unfavorable evaluations coexist. In contrast, bipolar fuzzy representations consider both positive and negative membership degrees, enabling a dual-perspective assessment. The LRBTFN further enhances this representation by allowing asymmetric spreads through left (L) and right (R) functions. This flexibility is particularly useful in modeling complex real-world problems, where uncertainty is often non-uniform due to criteria variability and subjective expert judgment. Therefore, a LRBTFN is defined in this work and it provides a more realistic and expressive tool for handling imprecision compared with traditional fuzzy models.

**Definition 3.2.** *A LRBTFN on the real line* ℝ *is defined as*


$$\begin{array}{l} A=\left(\left(m^{+},a^{+},b^{+}\right),\left(m^{-},a^{-},b^{-}\right)\right), \end{array}$$

*where the positive and negative membership functions* $\mu_{A}^{+}\left(x\right)$ *and* $\mu_{A}^{-}\left(x\right)$ *are defined as:*


$$\begin{array}{l} \begin{array}{c} \mu_{A}^{+}(x)=\{\begin{array}{cc} L^{+}\left(\frac{x-\left(m^{+}-a^{+}\right)}{a^{+}}\right), & if\;m^{+}-a^{+}\leq x\leq m^{+},\\ R^{+}\left(\frac{\left(m^{+}+b^{+}\right)-x}{b^{+}}\right), & if\;m^{+}\leq x\leq m^{+}+b^{+},\\ 0, & \;otherwise,\; \end{array}\\ \mu_{A}^{-}(x)=\{\begin{array}{cc} -L^{-}\left(\frac{x-\left(m^{-}-a^{-}\right)}{a^{-}}\right), & if\;m^{-}-a^{-}\leq x\leq m^{-},\\ -R^{-}\left(\frac{\left(m^{-}+b^{-}\right)-x}{b^{-}}\right), & if\;m^{-}\leq x\leq m^{-}+b^{-},\\ 0, & \;otherwise.\; \end{array} \end{array} \end{array}$$

**Figure 1:**
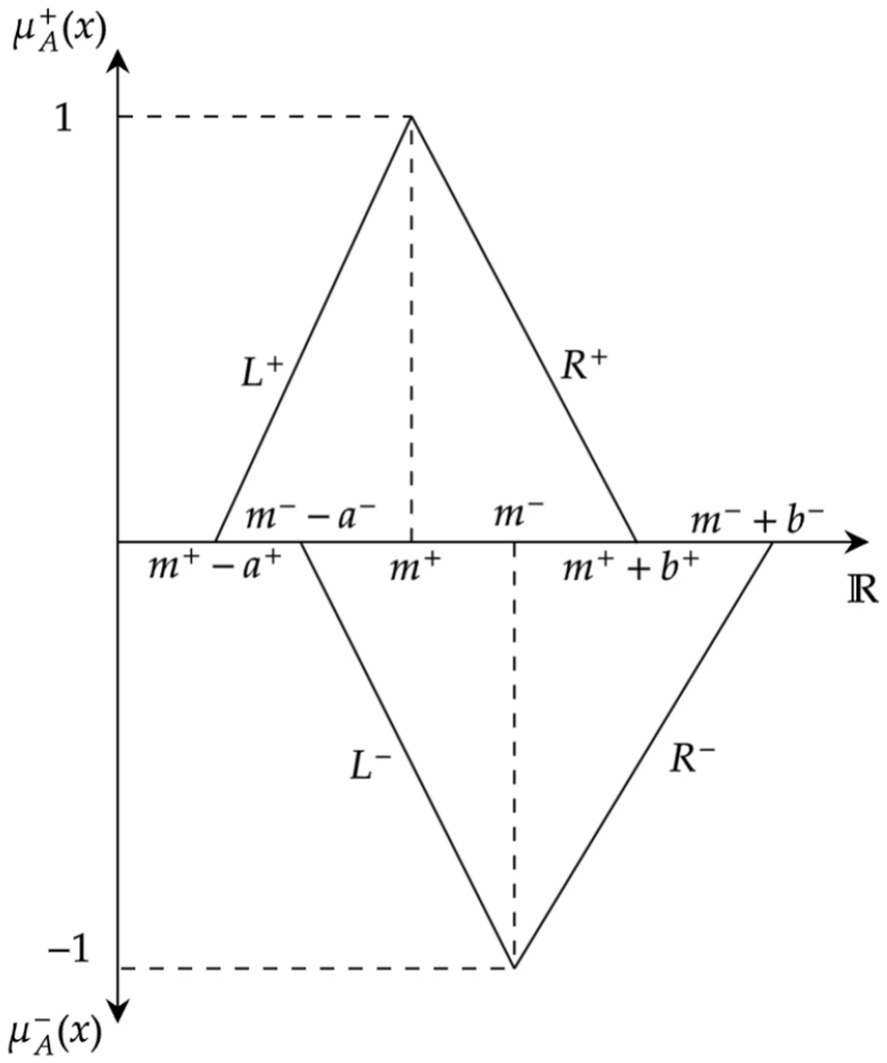
Representation of LRBTFN.

Here, *L* and *R* represent the left and right reference functions, respectively. The parameters *a*^+^, *b*^+^ and *a*^−^, *b*^−^ are the left and right spreads of the positive and negative membership functions. Figure 1 demonstrates the pictorial representation of the LRBTFN.

**Definition 3.3.** *Let* $A_{1}=\left(\left(m_{1}^{+},a_{1}^{+},b_{1}^{+}\right),\left(m_{1}^{-},a_{1}^{-},b_{1}^{-}\right)\right)$ *and* $A_{2}=\left(\left(m_{2}^{+},a_{2}^{+},b_{2}^{+}\right),\left(m_{2}^{-},a_{2}^{-},b_{2}^{-}\right)\right)$ *be any two LRBTFNs. Then, the arithmetic operations are defined as:*


$$\begin{array}{l} (i).\begin{array}{c} \;A_{1}+A_{2}=(\left(m_{1}^{+}+m_{2}^{+},a_{1}^{+}+a_{2}^{+},b_{1}^{+}+b_{2}^{+}\right),(m_{1}^{-}\\ +m_{2}^{-},a_{1}^{-}+a_{2}^{-},b_{1}^{-}+b_{2}^{-})), \end{array}\\ (ii).\begin{array}{c} A_{1}-A_{2}=(\left(m_{1}^{+}-m_{2}^{+},a_{1}^{+}+b_{2}^{+},b_{1}^{+}+a_{2}^{+}\right),(m_{1}^{-}\\ -m_{2}^{-},a_{1}^{-}+b_{2}^{-},b_{1}^{-}+a_{2}^{-})), \end{array}\\ (iii).\;-A_{2}=\left(\left(-m_{2}^{+},b_{2}^{+},a_{2}^{+}\right),\left(-m_{2}^{-},b_{2}^{-},a_{2}^{-}\right)\right),\\ (iv).\;\begin{array}{c} A_{1}*A_{2}=(\left(P^{+},P^{+}-\min\left(S^{+}\right),\max\left(S^{+}\right)-P^{+}\right),(P^{-},P^{-}\\ -\min\left(S^{-}\right),\max\left(S^{-}\right)-P^{-})), \end{array}\\ \begin{array}{c} \;where\;P^{+}=m_{1}^{+}*m_{2}^{+},S^{+}=\{(m_{1}^{+}-a_{1}^{+})*(m_{2}^{+}\\ -a_{2}^{+}),\left(m_{1}^{+}+b_{1}^{+}\right)*\left(m_{2}^{+}+b_{2}^{+}\right)\}, \end{array}\\ P^{-}=m_{1}^{-}*m_{2}^{-},S^{-}=\{(m_{1}^{-}-a_{1}^{-})*(m_{2}^{-}\\ -a_{2}^{-}),\left(m_{1}^{-}+b_{1}^{-}\right)*\left(m_{2}^{-}+b_{2}^{-}\right)\}\\ (v).\;\begin{array}{c} \frac{A_{1}}{A_{2}}=(\left(Q^{+},Q^{+}-\min\left(T^{+}\right),\max\left(T^{+}\right)-Q^{+}\right),(Q^{-},\\ Q^{-}-\min\left(T^{-}\right),\max\left(T^{-}\right)-Q^{-})), \end{array}\\ \;where\;Q^{+}=\frac{m_{1}^{+}}{m_{2}^{+}},S^{+}=\left\{\frac{\left(m_{1}^{+}-a_{1}^{+}\right)}{\left(m_{2}^{+}-a_{2}^{+}\right)},\frac{\left(m_{1}^{+}+b_{1}^{+}\right)}{\left(m_{2}^{+}+b_{2}^{+}\right)}\right\},\\ Q^{-}=\frac{m_{1}^{-}}{m_{2}^{-}},S^{-}=\left\{\frac{\left(m_{1}^{-}-a_{1}^{-}\right)}{\left(m_{2}^{-}-a_{2}^{-}\right)},\frac{\left(m_{1}^{-}+b_{1}^{-}\right)}{\left(m_{2}^{-}+b_{2}^{-}\right)}\right\}\\ (vi).\;\begin{array}{c} \;k*A_{1}=(\left(k*m_{1}^{+},|k|*a_{1}^{+},|k|*b_{1}^{+}\right),(k*m_{1}^{-},|k|\\ {}*a_{1}^{-},|k|*b_{1}^{-})),\;\;where\;k\in \mathbb{R} \end{array}\\ \begin{array}{c} A_{1}^{k}=(({\left(m_{1}^{+}\right)}^{k},{\left(m_{1}^{+}\right)}^{k}-{\left(m_{1}^{+}-a_{1}^{+}\right)}^{k},{\left(m_{1}^{+}+b_{1}^{+}\right)}^{k}\\ -{\left(m_{1}^{+}\right)}^{k}),\\ ({\left(m_{1}^{-}\right)}^{k},{\left(m_{1}^{-}\right)}^{k}-{\left(m_{1}^{-}-a_{1}^{-}\right)}^{k},{\left(m_{1}^{-}+b_{1}^{-}\right)}^{k}\\ -{\left(m_{1}^{-}\right)}^{k})),\;\;where\;k\in {\mathbb{R}}^{+} \end{array} \end{array}$$

**Definition 3.4.** *Let A* = ((*m*^+^, *a*^+^, *b*^+^), (*m*^−^, *a*^−^, *b*^−^)) *be a LRBTFN on* ℝ. *Then, a new ranking function R*(*A*) *is defined as:*


$$\begin{array}{l} R\left(A\right)=\frac{3+3m^{+}-3m^{-}+b^{+}-b^{-}-a^{+}+a^{-}}{6}. \end{array}$$

**Definition 3.5.** *Let A*_1_ *and A*_2_ *be two LRBTFNs with ranking values R*(*A*_1_) and *R*(*A*_2_) *respectively. Then the comparison rule is given by:*

*(i) A*_1_≻*A*_2_, if *R*(*A*_1_)>*R*(*A*_2_),
*(ii) A*_1_≺*A*_2_, if *R*(*A*_1_) < *R*(*A*_2_),
*(iii) A*_1_≈*A*_2_, if *R*(*A*_1_) = *R*(*A*_2_).

## 4 Methodology: a hybrid LR-bipolar triangular fuzzy PSI-SAW-CODAS model

In this part, the algorithm of the proposed hybrid BLRTF decision-making model is discussed.

### 4.1 Algorithm of the proposed model

**Stage 1 - Initialization process**

**Step 1: Frame the linguistic decision matrices (LDMs)**

The LDMs $L^{e}={\left[l_{ij}^{e}\right]}_{m\times n}$ are constructed with the help of *t* experts, where $l_{ij}^{e}$ represents the linguistic term for the *i*^*th*^ alternative and *j*^*th*^ criterion by the *e*^*th*^ expert.

**Step 2: Convert linguistic terms to LRBTFN decision matrices**

The LDMs *L*^*e*^ are transformed into LRBTFN decision matrices *D*^*e*^.


$$\begin{array}{l} d_{ij}^{e}=\left[\begin{array}{cccc} d_{11}^{e} & d_{12}^{e} & \ldots & d_{1n}^{e}\\ d_{21}^{e} & d_{22}^{e} & \ldots & d_{2n}^{e}\\ \vdots & \vdots & \ddots & \vdots \\ d_{m1}^{e} & d_{m2}^{e} & \ldots & d_{mn}^{e} \end{array}\right] \end{array}$$

where $d_{ij}^{e}=\left(\left(d_{ij}^{e+},da_{ij}^{e+},db_{ij}^{e+}\right),\left(d_{ij}^{e-},da_{ij}^{e-},db_{ij}^{e-}\right)\right)$ is the LRBTFN for the *i*^*th*^ alternative and *j*^*th*^ criterion as given by the *e*^*th*^ expert.

**Step 3: Normalize the LRBTFN decision matrices**

The normalized matrices $Y^{e}={\left[y_{ij}^{e}\right]}_{m\times n}$ are obtained using Equations 1 and 2 for beneficial and cost criteria, respectively.

For benefit criteria:


$$\begin{array}{l} y_{ij}^{e}=\left(\frac{\left(d_{ij}^{e+},da_{ij}^{e+},db_{ij}^{e+}\right)}{d_{\max j}^{e+}+db_{\max j}^{e+}},\frac{\left(d_{ij}^{e-},da_{ij}^{e-},db_{ij}^{e-}\right)}{d_{\max j}^{e-}+db_{\max j}^{e-}}\right) \end{array}$$ (1)

For cost criteria:


$$\begin{array}{l} y_{ij}^{e}=\left(\frac{d_{\min j}^{e+}-da_{\min j}^{e+}}{\left(d_{ij}^{e+},da_{ij}^{e+},db_{ij}^{e+}\right)},\frac{d_{\min j}^{e-}-da_{\min j}^{e-}}{\left(d_{ij}^{e-},da_{ij}^{e-},db_{ij}^{e-}\right)}\right) \end{array}$$ (2)

where $\left(\left(y_{ij}^{e+},ya_{ij}^{e+},yb_{ij}^{e+}\right),\left(y_{ij}^{e-},ya_{ij}^{e-},yb_{ij}^{e-}\right)\right)$.

**Stage 2 - PSI process to find objective weights**

**Step 4: Compute the preference selection index**

The preference selection index values $p_{j}^{e}$ are calculated for each criterion using Equation 3.


$$\begin{array}{l} p_{j}^{e}=\overset{m}{\underset{i=1}{\sum }}{\left(y_{ij}^{*e}-{ȳ}_{j}^{*e}\right)}^{2}, \end{array}$$ (3)

where $y_{ij}^{*e}=\frac{3+3y_{ij}^{e+}-y_{ij}^{e-}+ya_{ij}^{e+}-ya_{ij}^{e-}-yb_{ij}^{e+}+yb_{ij}^{e-}}{6}$, ${ȳ}_{j}^{*e}=\frac{1}{n}\overset{n}{\underset{i=1}{\sum }}y_{ij}^{*e}$ and$p_{j}^{e}=\left(\left(p_{j}^{e+},pa_{j}^{e+},pb_{j}^{e+}\right),\left(p_{j}^{e-},pa_{j}^{e-},pb_{j}^{e-}\right)\right)$.

**Step 5: Determine the overall preference weight vector**

The weight vectors $W={\left[w_{j}^{e}\right]}_{1\times n}$ are computed using Equation 4.


$$\begin{array}{l} w_{j}^{e}=\frac{\left(1-p_{j}^{e}\right)*z_{e}}{\overset{n}{\underset{j=1}{\sum }}1-p_{j}^{e}} \end{array}$$ (4)

where $w_{j}^{e}=\left(\left(w_{j}^{e+},wa_{j}^{e+},wb_{j}^{e+}\right),\left(w_{j}^{e-},wa_{j}^{e-},wb_{j}^{e-}\right)\right)$ and *z*_*e*_ represents the preference weight for *e*^*th*^ decision expert.

**Stage 3 - SAW process to aggregate the weight and decision matrices**

**Step 6: Aggregate the decision matrices with weights**

The decision matrices and the weight vectors are aggregated to obtain the aggregated information using Equation 5.


$$\begin{array}{l} D={\left[x_{ij}\right]}_{m\times n}=\frac{1}{n}\overset{t}{\underset{e=1}{\sum }}y_{ij}^{e}*w_{j}^{e} \end{array}$$ (5)

where $x_{ij}=(\left(w_{j}^{e+}*y_{ij}^{e+},\left|wa_{j}^{e+}\right|*ya_{ij}^{e+},\left|wb_{j}^{e+}\right|*yb_{ij}^{e+}\right),(w_{j}^{e-}*$ $y_{ij}^{e-},\left|wa_{j}^{e-}\right|*ya_{ij}^{e-},\left|wb_{j}^{e-}\right|*yb_{ij}^{e-}))$ $=\left(\left(x_{ij}^{+},xa_{ij}^{+},xb_{ij}^{+}\right),\left(x_{ij}^{-},xa_{ij}^{-},xb_{ij}^{-}\right)\right)$

**Stage 4 - CODAS process to compute the assessment score**

**Step 7: Calculate utility scores through Euclidean and Hamming distance measures**

The utility scores of each alternative are determined by applying Euclidean (*E*_*i*_) and Hamming (*H*_*i*_) distances between the aggregated decision scores and negative ideal solutions of the alternative using Equations 6 and 7.


$$\begin{array}{l} E_{i}=\sum_{j=1}^{n}\sqrt{\begin{array}{c} ({\left(x_{ij}^{+}-x_{\min j}^{+}\right)}^{2}+{\left(xa_{ij}^{+}-xa_{\min j}^{+}\right)}^{2}+{\left(xb_{ij}^{+}-xb_{\min j}^{+}\right)}^{2}\\ +{\left(x_{ij}^{-}-x_{\min j}^{-}\right)}^{2}+{\left(xa_{ij}^{-}-xa_{\min j}^{-}\right)}^{2}+(xb_{ij}^{-}\\ {-xb_{\min j}^{-})}^{2} \end{array}} \end{array}$$ (6)

$$\begin{array}{l} H_{i}=\sum_{j=1}^{n}\sqrt{\begin{array}{c} (\left|x_{ij}^{+}-x_{\min j}^{+}\right|+\left|xa_{ij}^{+}-xa_{\min j}^{+}\right|+\left|xb_{ij}^{+}-xb_{\min j}^{+}\right|\\ +\left|x_{ij}^{-}-x_{\min j}^{-}\right|+\left|xa_{ij}^{-}-xa_{\min j}^{-}\right|+\left|xb_{ij}^{-}-xb_{\min j}^{-}\right| \end{array})} \end{array}$$ (7)

where $x_{j}^{*}=\left(\left(x_{\min j}^{+},xa_{\min j}^{+},xb_{\min j}^{+}\right),\left(x_{\min j}^{-},xa_{\min j}^{-},xb_{\min j}^{-}\right)\right)$ is the negative ideal value of the *j*^*th*^ criterion.

**Step 8: Construct the relative assessment matrix R**

The relative assessment matrix *R* is obtained using Equation 8.


$$\begin{array}{l} R={\left[r_{ij}\right]}_{n\times n}=\left(E_{i}-E_{j}\right)+\left(\lambda \left(E_{i}-E_{j}\right)\times \left(H_{i}-H_{j}\right)\right) \end{array}$$ (8)

where λ is a threshold function to recognize the equality of the Euclidean distances of two alternatives based on the threshold parameter *c* = 0.02 (Ghorabaee et al., 2016) and is defined as follows:


$$\begin{array}{l} \lambda =\{\begin{array}{ll} 1 & E_{i}-E_{j}\geq c\\ 0 & E_{i}-E_{j}<c \end{array} \end{array}$$

**Step 9: Compute the assessment score *R*_*i*_ for each alternative**.

The assessment scores *R*_*i*_ for each alternative can be calculated using Equation 9.


$$\begin{array}{l} R_{i}=\overset{n}{\underset{j=1}{\sum }}r_{ij},\;i=1,2,\ldots ,m. \end{array}$$ (9)

**Stage 5 - Ranking process**

**Step 10: Rank the alternatives**.

The alternatives are ranked by arranging the assessment score in descending order.

**Remark:** The defuzzification process is only applied weight assessment through PSI method based on defined score function. Unlike many fuzzy MCDM approaches, the proposed PSI–SAW–CODAS framework does not require an explicit defuzzification step before ranking process. The ranking of alternatives is obtained directly by the computation of Euclidean and Hamming distance measures under LRBTFN environment. These distance measures transform the fuzzy information into scalar utility scores by evaluating the deviation of each alternative from the negative ideal solution. As a result, the need for an additional defuzzification function is eliminated. This characteristic provides a significant advantage, as it preserves the inherent uncertainty and avoids information loss that may occur during defuzzification. Further, the final ranking reflects a more reliable representation of the underlying fuzzy information.

***Justification of normalization scheme:*** The normalization process plays a crucial role in ensuring comparability among heterogeneous criteria. In this study, the normalization approach is specifically designed for LRBTFNs, preserving both their structural properties and uncertainty representation. Unlike conventional normalization techniques such as vector normalization, linear scaling, or max–min transformation, the proposed method operates directly on the fuzzy parameters of both positive and negative components. This ensures that the inherent fuzziness and bipolar nature of the data are not distorted during scaling. For benefit criteria, the normalization is performed using the maximum upper bound of the fuzzy values, which guarantees proportional scaling while maintaining the relative performance of alternatives. For cost criteria, the transformation is based on the minimum lower bound, effectively converting them into benefit-type measures without altering their semantic interpretation.

This dual normalization strategy ensures:

- consistency between benefit and cost attributes,
- preservation of uncertainty information,
- avoidance of scale distortion across criteria, and
- compatibility with developed distance-based evaluation.

Therefore, the adopted normalization method is more suitable for bipolar fuzzy environments compared to classical normalization approaches.

**Figure 2:**
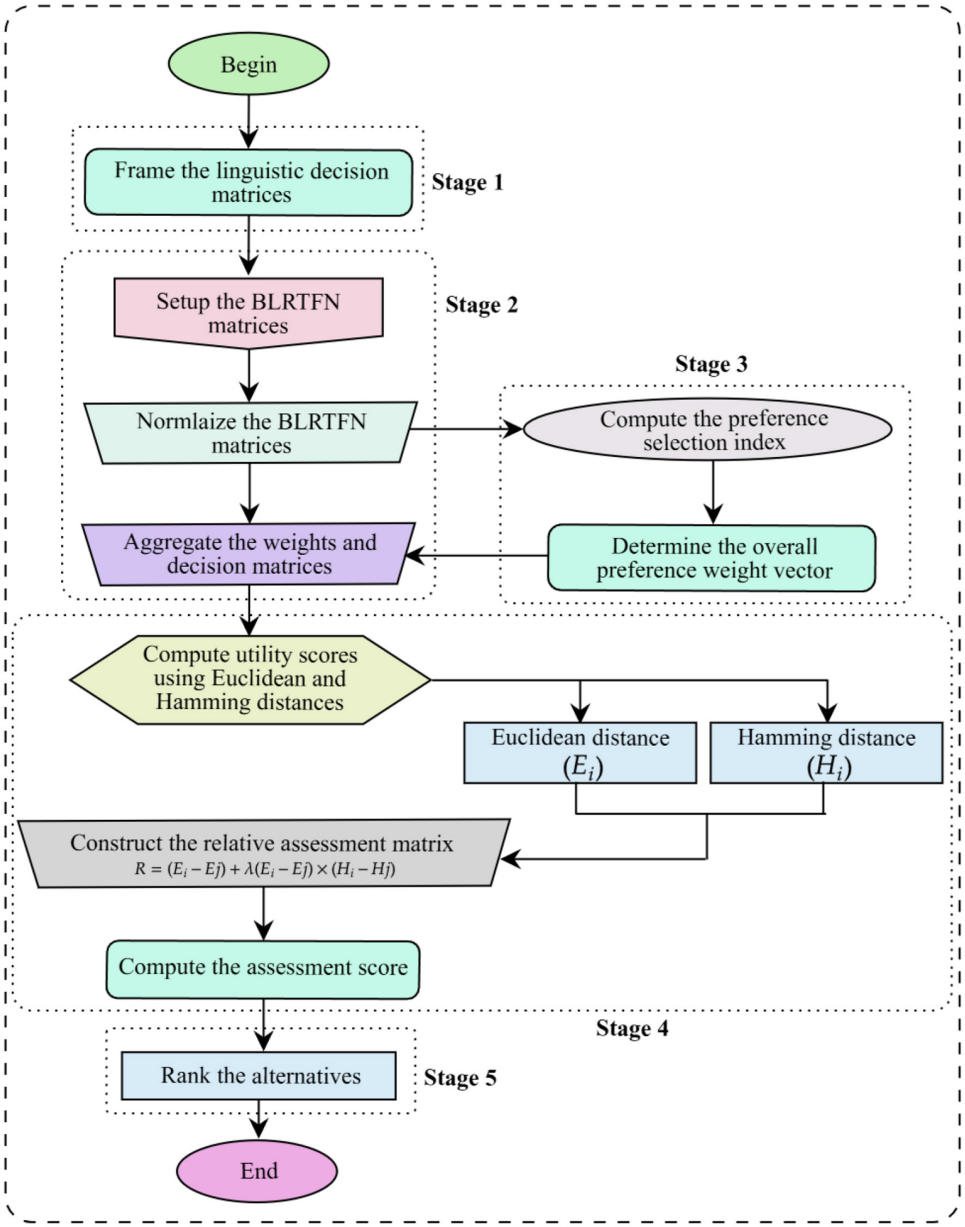
Workflow of the proposed model.

A simplified workflow is provided in Algorithm 1 to briefly discuss the step-by-step procedure of the proposed model. Also, a flowchart of the proposed model is depicted in Figure 2.

Algorithm 1: **Simplified workflow of the proposed PSI–SAW–CODAS model under LRBTFN environment.**

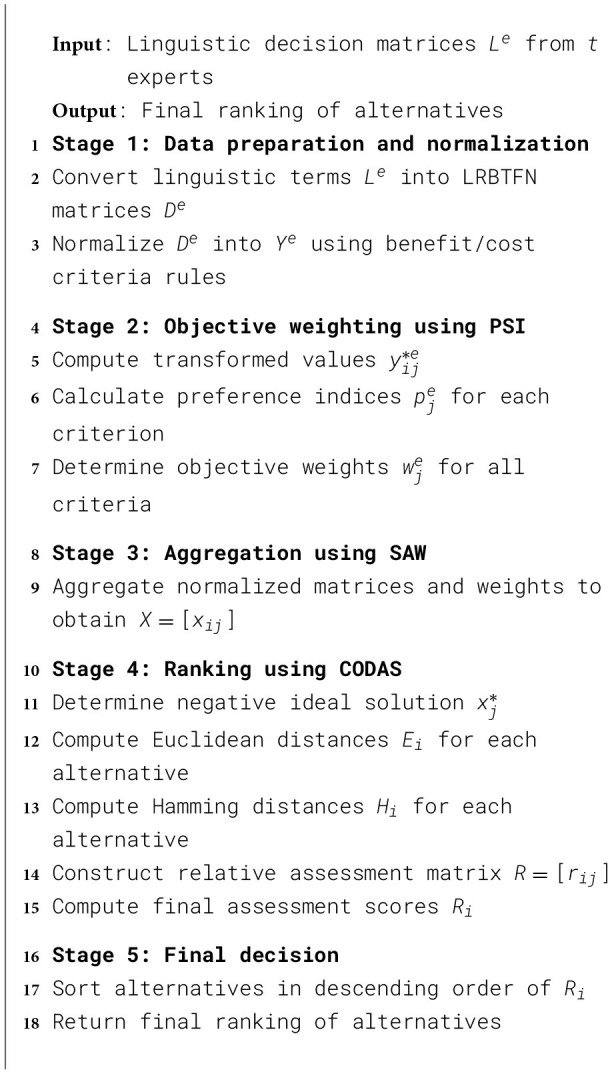

## 5 Case study: identifying flood-prone zone in Ernakulam district, Kerala

### 5.1 Study context

The present study focuses on identifying and prioritizing flood-prone areas within the Ernakulam district, one of the most urbanized and densely populated districts in the Indian state of Kerala. Geographically located along the southwest coast of India, Ernakulam is bordered by the Arabian Sea to the west and is traversed by numerous rivers and backwaters, including the Periyar, Muvattupuzha, and Chalakkudy rivers. These hydrological features, combined with high monsoonal rainfall and rapid urban expansion, make the district highly susceptible to flooding, especially during the southwest monsoon season.

**Figure 3:**
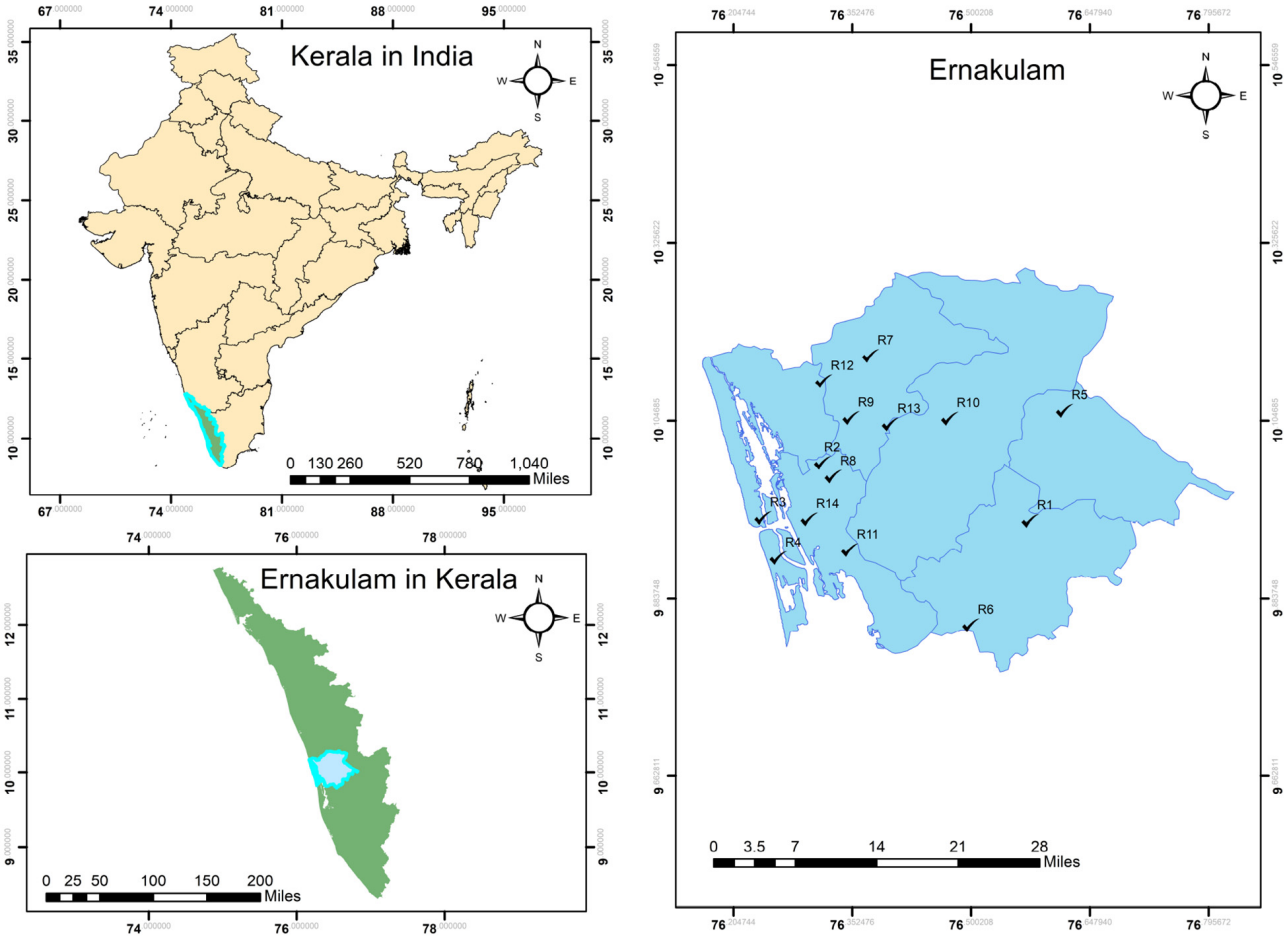
Study area of the research.

Figure 3 shows the GIS representation of Kerala highlighting the Ernakulam district and the 14 critical regions considered for flood vulnerability assessment. This spatial context facilitates understanding the geographical diversity and hydrological features influencing flood risks in the area.

In this study, 14 critical regions within the district, namely Muvattupuzha (*R*_1_), Kalamassery (*R*_2_), Vypen (*R*_3_), Kochi (*R*_4_), Kothamangalam (*R*_5_), Piravom (*R*_6_), Angamaly (*R*_7_), Thrikkakara (*R*_8_), Aluva (*R*_9_), Perumbavoor (*R*_10_), Thripunithura (*R*_11_), Paravoor (*R*_12_), Kunnathunad (*R*_13_), and Ernakulam (*R*_14_), are considered as the decision alternatives. These areas vary significantly in terms of elevation, proximity to water bodies, and degree of urbanization, thus providing a diverse landscape for flood vulnerability assessment.

### 5.2 Selection and justification of criteria

To comprehensively evaluate flood susceptibility, ten key criteria have been selected based on their established relevance in the literature, expert consultation, and the specific geographical and environmental characteristics of the Ernakulam district. These criteria include: (*C*_1_) altitude, (*C*_2_) historical flood records, (*C*_3_) rainfall intensity, (*C*_4_) soil type, (*C*_5_) land use and urbanization patterns, (*C*_6_) ground slope, (*C*_7_) existing flood control measures, (*C*_8_) proximity to water bodies, (*C*_9_) population density, and (*C*_10_) green cover. Collectively, these criteria represent topographical, hydrological, environmental, and socio-economic dimensions of flood vulnerability.

Topographical factors such as altitude and ground slope play a fundamental role in determining water accumulation and flow dynamics. Low-lying areas and regions with gentle slopes are more susceptible to water stagnation, thereby increasing flood risk. Hydrological factors, including rainfall intensity and proximity to water bodies, directly influence the volume and movement of water during extreme weather events. High rainfall leads to excessive runoff, while areas located near rivers, backwaters, and coastal zones are more prone to inundation. Environmental characteristics such as soil type and green cover significantly affect infiltration capacity and surface runoff. Soils with low permeability reduce water absorption, leading to higher runoff, whereas dense vegetation enhances infiltration and mitigates flood impact. On the other hand, anthropogenic factors, including land use, urbanization, and population density, contribute to altered drainage patterns and increased surface impermeability. Rapid urban expansion and high population concentration often result in inadequate drainage infrastructure, thereby intensifying flood vulnerability. Additionally, the presence or absence of effective flood control measures plays a critical role in mitigating or exacerbating flood impacts. Historical flood records provide valuable insights into the recurrence and severity of past flood events, thereby serving as an important indicator of flood-prone regions. The integration of these diverse yet interrelated criteria ensures a holistic assessment of flood susceptibility by capturing both natural processes and human-induced influences.

Therefore, the selected criteria not only reflect the complex interplay of physical and socio-environmental factors but also enhance the robustness and practical relevance of the proposed hybrid LR-bipolar triangular fuzzy decision-making model for prioritizing flood-prone zones in the Ernakulam district.

### 5.3 Experts' assessments and linguistic decisions

Experts' assessment: The prioritization of flood-prone zones in the Ernakulam district, Kerala, was carried out with the support of four experts in various domain possessing significant academic and professional expertise. The expert panel includes academicians and industry practitioners specializing in areas such as disaster management, environmental engineering, hydraulic engineering, cloud computing, and instrumentation engineering. All experts have more than ten years of experience in their respective domains, with direct or indirect involvement in flood-related studies, environmental analysis, or infrastructure systems.

The selection of experts was based on their domain knowledge, research contributions, and practical exposure to flood risk assessment and management. This ensured that both theoretical understanding and real-world insights were incorporated into the evaluation process. The experts were requested to provide their judgments independently using a predefined linguistic scale, thereby minimizing bias and ensuring consistency in the evaluation. Their assessments were utilized to evaluate the relative importance of the selected criteria and the performance of each alternative. The integration of these expert opinions within the LRBTFN framework further enhances the reliability of the decision-making process by effectively handling uncertainty and vagueness in human judgments.

### 5.4 Inter-criteria dependency analysis

In flood susceptibility assessment, certain conditioning factors such as rainfall, slope, land use, and proximity to water bodies may exhibit inherent correlations. For instance, areas with steep slopes may influence runoff patterns, while urbanization may affect drainage capacity. In the present study, the criteria are treated as independent, which is a common assumption in many MCDM-based approaches. However, the potential influence of inter-criteria relationships is indirectly accounted for through the adopted methodology. The PSI-based weighting scheme evaluates the dispersion of criterion values across alternatives, which helps mitigate redundancy effects arising from correlated variables. Additionally, expert judgments reflect practical knowledge of the domain, thereby incorporating implicit understanding of such relationships. Although explicit statistical techniques such as correlation analysis or multicollinearity testing are not employed in this study, the obtained results show strong agreement with real-world flood patterns.

These qualitative assessments were then used to form individual linguistic decision matrices for each expert, denoted as $L^{e}={\left[l_{ij}^{e}\right]}_{m\times n}$, where $l_{ij}^{e}$ represents the linguistic rating of the *i*^th^ alternative with respect to the *j*^th^ criterion given by the *e*^th^ expert. The linguistic scale for LRBTFN is provided in Table 1 and the linguistic matrix given in Table 2 provides the collection of information.

**Table 1:** Linguistic scale.

Linguistic term	LR-bipolar triangular fuzzy number
Extremely Low (EL)	(0.05, 0, 0.1), (0.95, 0.05, 0)
Very Low (VL)	(0.1, 0.05, 0.05), (0.9, 0.1, 0.05)
Low (L)	(0.2, 0.1, 0.05), (0.8, 0.05, 0.1)
Medium Low (ML)	(0.3, 0.1, 0.05), (0.7, 0.05, 0.1)
Medium (M)	(0.5, 0.1, 0.1), (0.5, 0.1, 0.1)
Medium High (MH)	(0.7, 0.05, 0.1), (0.3, 0.1, 0.05)
High (H)	(0.8, 0.1, 0.05), (0.2, 0.05, 0.1)
Very High (VH)	(0.9, 0.05, 0.1), (0.1, 0.05, 0.05)
Extremely High (EH)	(0.95, 0.1, 0), (0.05, 0, 0.1)

**Table 2:** Linguistic information from the experts.

Alternative	*C*_1_	*C*_2_	*C*_3_	*C*_4_	*C*_5_
Muvattupuzha	(M, MH, MH, ML)	(H, M, M, L)	(MH, M, M, VL)	(M, M, M, M)	(H, H, H, EH)
Kalamassery	(ML, VL, VL, VL)	(EH, EH, EH, VH)	(MH, M, M, L)	(L, M, M, M)	(MH, M, M, H)
Vypen	(MH, EL, EL, EL)	(H, VH, VH, M)	(H, M, M, L)	(ML, VL, VL, L)	(H, L, L, VH)
Kochi	(L, EL, EL, L)	(VH, H, H, M)	(VH, M, M, M)	(L, L, L, L)	(VH, VH, VH, EH)
Kothamangalam	(L, ML, ML, M)	(MH, M, M, L)	(H, M, M, M)	(H, M, M, M)	(H, M, M, H)
Piravom	(VL, L, L, ML)	(H, H, H, M)	(VH, ML, ML, L)	(L, L, L, L)	(VH, H, H, VH)
Angamaly	(M, ML, ML, MH)	(H, L, L, L)	(VH, M, M, L)	(M, M, M, MH)	(VH, H, H, H)
Thrikkakara	(H, M, M, H)	(MH, L, L, L)	(H, MH, MH, H)	(M, MH, MH, H)	(H, M, M, MH)
Aluva	(M, L, L, MH)	(EH, H, H, VH)	(VH, M, M, M)	(M, M, M, H)	(H, ML, ML, H)
Perumbavoor	(MH, M, M, H)	(MH, L, L, L)	(H, MH, MH, M)	(M, MH, MH, H)	(MH, M, M, MH)
Thripunithura	(H, M, M, VH)	(M, L, L, EL)	(MH, M, M, M)	(H, MH, MH, M)	(M, M, M, ML)
Paravoor	(H, M, M, VH)	(M, L, L, EL)	(MH, M, M, MH)	(H, MH, MH, VH)	(M, M, M, ML)
Kunnathunad	(H, MH, MH, EH)	(ML, L, L, L)	(M, MH, MH, H)	(MH, MH, MH, EH)	(H, M, M, MH)
Ernakulam	(VH, H, H, H)	(ML, L, L, L)	(M, H, H, H)	(H, H, H, H)	(M, M, M, ML)
**Alternative**	***C*_6_**	***C*_7_**	***C*_8_**	***C*_9_**	***C*_10_**
Muvattupuzha	(M, M, M, L)	(H, H, H, M)	(M, M, M, L)	(VH, M, M, H)	(M, M, M, L)
Kalamassery	(MH, L, L, L)	(H, L, L, L)	(VH, H, H, H)	(H, M, M, EH)	(H, M, M, MH)
Vypen	(M, L, L, VL)	(MH, L, L, L)	(VH, VH, VH, EH)	(H, VH, VH, EH)	(MH, M, M, VL)
Kochi	(L, L, L, VL)	(H, M, M, ML)	(VH, VH, VH, EH)	(VH, VH, VH, VH)	(L, M, M, VL)
Kothamangalam	(M, M, M, M)	(H, M, M, M)	(MH, M, M, M)	(H, M, M, M)	(M, MH, MH, M)
Piravom	(M, L, L, ML)	(VH, M, M, ML)	(VH, H, H, MH)	(VH, H, H, VH)	(L, M, M, VL)
Angamaly	(MH, M, M, M)	(MH, M, M, H)	(MH, M, M, L)	(H, MH, MH, MH)	(M, M, M, H)
Thrikkakara	(H, MH, MH, MH)	(VH, M, M, MH)	(L, M, M, ML)	(H, MH, MH, MH)	(H, MH, MH, VH)
Aluva	(MH, M, M, MH)	(VH, M, M, ML)	(VH, VH, VH, VH)	(VH, H, H, H)	(M, MH, MH, M)
Perumbavoor	(H, M, M, H)	(MH, M, M, H)	(MH, M, M, L)	(M, M, M, H)	(M, MH, MH, MH)
Thripunithura	(MH, M, M, VH)	(H, M, M, VH)	(M, M, M, EL)	(ML, M, M, MH)	(H, M, M, VH)
Paravoor	(MH, M, M, EH)	(M, M, M, VH)	(H, M, M, L)	(ML, M, M, MH)	(H, M, M, VH)
Kunnathunad	(M, MH, MH, EH)	(MH, MH, MH, VH)	(H, H, H, ML)	(M, M, M, H)	(H, H, H, H)
Ernakulam	(ML, H, H, VH)	(M, M, M, H)	(M, M, M, L)	(ML, M, M, MH)	(VH, H, H, VH)

### 5.5 Implementation of the model

The LRBTFNs are applied in the proposed decision-making model to obtain the ranking order based on the significant criteria.

In stage 1, the process of collecting the linguistic matrices and their transformations is performed in steps 1 and 2, respectively. In Appendix A, the decision matrices with respect to four experts are provided in Tables A1–A4 in Appendix A. In step 3, the normalized decision matrices are obtained using Equations 1 and 2. In stage 2, the PIV method is processed to determine the weights of the criteria. The preference selection index for all the criteria is obtained using Equation 3 in step 4. In step 5, the weight of each criterion is computed using Equation 4. In this process, the preference weight for the experts is also considered as *z*_1_ = 0.3, *z*_2_ = 0.15, *z*_3_ = 0.2 and *z*_4_ = 0.35 based on their experience and consensus. Then, stage 3 is processed to aggregate the decision matrices and weights of the criteria using the SAW method. In step 6, the weights of the criteria and decision matrices are aggregated using Equation 5. In step 7, the utility scores are obtained by applying the LR-bipolar triangular fuzzy Euclidean and Hamming distances (Table 3) between the aggregated decision matrix and the negative ideal solutions of the criteria. In step 8, the relative assessment matrix is constructed using Equation 8, where the threshold parameter *c* = 0.02 is considered to fix the threshold function λ. In step 9, the assessment score for each alternative is computed using Equation 9. Finally, the rank (Table 3) of the alternative is assigned by arranging the idealized assessment scores in descending order.

**Table 3:** Euclidean, hamming distances, ideal scores and rank.

*R*_*i*_	*E*_*i*_	*E*_*i*_	Score	Rank
*R*_1_	0.1196	0.0292	0.3677	13
*R*_2_	0.1199	0.0416	0.6695	8
*R*_3_	0.1349	0.0110	0.8010	2
*R*_4_	0.1570	0.0220	1.0000	1
*R*_5_	0.0794	0.0125	0.3101	14
*R*_6_	0.1239	0.0342	0.7055	7
*R*_7_	0.0958	0.0268	0.4526	11
*R*_8_	0.1111	0.0308	0.5902	9
*R*_9_	0.1257	0.0474	0.7228	5
*R*_10_	0.0901	0.0144	0.4043	12
*R*_11_	0.1247	0.0365	0.7131	6
*R*_12_	0.1279	0.0420	0.7421	3
*R*_13_	0.1056	0.0459	0.5385	10
*R*_14_	0.1263	0.0597	0.7288	4

## 6 Results and discussion

The proposed hybrid PSI–SAW–CODAS model under the LRBTFN environment was successfully implemented to identify the most flood-prone zones in the Ernakulam district of Kerala. A total of 14 alternatives representing specific geographical regions were evaluated using 10 vital criteria, including altitude, historical flood data, rainfall, soil type, land use and urbanization, ground slope, flood control measures, proximity to water bodies, population density, and green cover. The linguistic judgments were obtained from four domain experts familiar with the flood behavior and environmental settings in the region. These linguistic inputs were converted into LR-BTFNs and systematically processed through normalization, weighting, aggregation, and final assessment using the CODAS technique.

The model yielded a final rank order of the regions based on their susceptibility to flooding. The most flood-prone area was identified as Kochi, followed by Vypen, Paravoor, and Ernakulam. The least flood-prone region was determined to be Kothamangalam. The complete rank order from highest to lowest flood vulnerability is: Kochi (*R*_4_), Vypen (*R*_3_), Paravoor (*R*_12_), Ernakulam (*R*_14_), Aluva (*R*_9_), Thripunithura (*R*_11_), Piravom (*R*_6_), Kalamassery (*R*_2_), Thrikkakara (*R*_8_), Kunnathunadu (*R*_13_), Angamaly (*R*_7_), Perumbavoor (*R*_10_), Muvattupuzha (*R*_1_), and Kothamangalam (*R*_5_). These results highlight the interplay of natural and anthropogenic factors, such as low elevation, coastal and backwater proximity, dense population, and rapid urbanization, which elevate the flood risk in certain areas.

To further validate the practical applicability of the proposed model, the obtained ranking results are compared with historical flood events reported in the Ernakulam district. Notably, regions such as Kochi, Vypen, and Paravoor, which are identified among the most flood-prone zones in this study, have been severely affected during major flood events, particularly the Kerala floods of 2018, 2019, and 2023. These regions are characterized by low-lying terrain, proximity to backwaters and the Arabian Sea, and high urban density, all of which contribute to their repeated exposure to flooding. For instance, Kochi, ranked first in the proposed model, has consistently experienced urban flooding due to poor drainage systems, tidal influences, and heavy rainfall during monsoon seasons. Similarly, Vypen and Paravoor have been frequently inundated due to their coastal location and proximity to interconnected water bodies. These observations are well-documented in reports by government agencies and disaster management authorities, thereby supporting the reliability of the proposed ranking outcomes.

On the other hand, regions such as Kothamangalam and Muvattupuzha, which are ranked lower in terms of flood susceptibility, are relatively less affected due to higher elevation and better natural drainage conditions. This further demonstrates that the proposed model effectively captures both high-risk and low-risk zones in alignment with real-world flood patterns. Thus, the strong agreement between the model results and historical flood occurrences confirms the practical validity and robustness of the proposed PSI–SAW–CODAS framework under the LRBTFN environment.

### 6.1 Comparison with machine learning approaches

The proposed model is developed as a MCDM framework for prioritizing flood-prone regions based on multiple conflicting criteria and expert knowledge. Unlike machine learning models such as Random Forest, Gradient Boosting, or Neural Networks, which are primarily designed for prediction and classification using large-scale labeled datasets, the present approach focuses on structured decision analysis under uncertainty. Therefore, a direct comparison between the proposed MCDM model and machine learning algorithms may not be strictly appropriate, as they address different problem objectives. While machine learning models aim to predict flood occurrences, the proposed method aims to rank and prioritize regions based on multiple influencing factors.

However, machine learning techniques can complement MCDM approaches.

Interestingly, regions such as Kothamangalam, Perumbavoor, and Muvattupuzha, which are situated at higher altitudes and farther from coastal or riverine floodplains, ranked lower in flood susceptibility. This is consistent with their historical flood records, which show comparatively limited impacts. Thripunithura and Piravom, although urbanized, received intermediate rankings, reflecting their mixed vulnerability due to both terrain advantages and ongoing urban expansion. The integration of expert knowledge ensured that local context, infrastructural changes, and community preparedness were incorporated into the model, supplementing objective criteria with field-based insights. The hybrid structure of the model contributes significantly to its performance. The PSI method objectively determines the weights of criteria, the SAW method aggregates the weighted normalized decision matrix within the fuzzy environment, and CODAS ensures a discriminative final ranking by calculating Euclidean and Taxicab distances from the negative ideal solution. This layered approach provides a more realistic prioritization compared to conventional models.

The effectiveness of the proposed approach lies in its ability to closely mirror real-time scenarios. For instance, the identification of Kochi, Vypen, and Paravoor as the top three flood-prone regions strongly aligns with official reports from the Kerala State Disaster Management Authority (KSDMA) and recent flood events. The bipolar fuzzy framework effectively incorporates both positive and negative evaluations, capturing uncertainties and yielding results that are not only mathematically robust but also contextually reliable.

In conclusion, the proposed hybrid LRBTFN PSI–SAW–CODAS framework provides a comprehensive, systematic, and adaptable approach for flood-prone zone prioritization. Its ability to align closely with real-world flood behavior demonstrates its potential as a decision-making tool for planners, policymakers, and disaster management authorities in Kerala and beyond.

### 6.2 Comparative analysis

This section presents the outcomes of the flood vulnerability assessment conducted for 14 key regions within the Ernakulam district, Kerala. The prioritization was performed using the proposed hybrid bipolar LR-bipolar triangular fuzzy model. The study aims to analyze the variation and agreement among these methods in identifying flood-prone zones. Additionally, unsupervised machine learning techniques, namely K-means clustering and PCA, were employed to classify regions and uncover inherent patterns in vulnerability based on the MCDM outputs.

To validate the robustness and practical relevance of the proposed hybrid BLRTF decision-making model, a comparative analysis was conducted with four widely adopted MCDM approaches: TOPSIS, ARAS, MABAC, and MAUT using the constructed decision matrix. These methods were chosen as benchmarks since they are among the most established and well-recognized tools for ranking alternatives in multi-criteria contexts. Specifically, TOPSIS is frequently applied for problems requiring closeness to the ideal solution, ARAS emphasizes additive ratios and utility, MABAC is known for its balance area concept, and MAUT offers a classical perspective rooted in utility theory. By comparing with this diverse set of models, the evaluation covers both distance-based and utility-based perspectives, thereby ensuring fairness and comprehensiveness of the analysis.

Table 4 presents the scores and rankings obtained from each method. It can be observed that all approaches consistently identify Kochi as the top-ranked alternative, which aligns with ground realities since Kochi is historically the most flood-prone area within the district. Similarly, Vypen and Paravoor appear consistently within the top five in most methods, reflecting their high vulnerability due to coastal exposure and frequent inundations. However, considerable discrepancies are visible in the mid- and lower-ranked alternatives. For instance, ARAS ranks Kalamassery as the third most flood-prone zone, whereas the proposed method places it at the eighth position, which is more consistent with recent flood records. Likewise, Thripunithura receives a very low rank (14) in ARAS but is placed sixth by the proposed model, better reflecting the actual severity experienced during the 2018 and 2019 floods.

**Table 4:** Comparative study of flood-prone zone prioritization.

*R*_*i*_	TOPSIS		ARAS		MABAC		MAUT		Proposed	
	Score	Rank	Score	Rank	Score	Rank	Score	Rank	Score	Rank
*R*_1_	0.8414	7	0.9719	7	0.2793	12	0.4774	12	0.3677	13
*R*_2_	0.8141	10	0.9878	3	0.7096	3	0.7814	4	0.6695	8
*R*_3_	0.9485	2	0.9893	2	0.4697	5	0.7956	3	0.8010	2
*R*_4_	1.0000	1	1.0000	1	0.8376	2	1.0000	1	1.0000	1
*R*_5_	0.7236	14	0.9712	8	0.4152	6	0.7178	5	0.3101	14
*R*_6_	0.9355	3	0.9856	4	0.4144	7	0.6161	8	0.7055	7
*R*_7_	0.8214	9	0.9738	6	0.3737	9	0.6024	9	0.4526	11
*R*_8_	0.8112	11	0.9637	10	0.4895	4	0.7142	6	0.5902	9
*R*_9_	0.8512	6	0.9804	5	1.0000	1	0.8818	2	0.7228	5
*R*_10_	0.7619	13	0.9650	9	0.4071	8	0.7035	7	0.4043	12
*R*_11_	0.8260	8	0.9574	14	0.2500	14	0.4343	14	0.7131	6
*R*_12_	0.8562	5	0.9599	13	0.2691	13	0.4446	13	0.7421	3
*R*_13_	0.8023	12	0.9631	11	0.3248	11	0.5067	11	0.5385	10
*R*_14_	0.8922	4	0.9619	12	0.3578	10	0.5170	10	0.7288	4

All five methods were used to compute vulnerability scores for the 14 regions, considering 10 flood-related criteria. The scores and ranks derived from each method show both commonalities and variations. Across all methods, Kochi (*R*_4_) was unanimously ranked as the most vulnerable to flooding. This consistency is expected, given Kochi's low-lying terrain, proximity to the Arabian Sea, dense population, and extensive urban infrastructure. Likewise, Vypen (*R*_3_) and Aluva (*R*_9_) consistently appeared among the top five high-priority regions. These areas are situated near major rivers or coastal zones, making them naturally susceptible to water accumulation and overflow during monsoons. Some regions, such as Kalamassery (*R*_2_) and Thripunithura (*R*_11_), showed noticeable rank fluctuations across methods. For instance, while ARAS ranked R2 as third, TOPSIS placed it at the tenth position. Such differences arise due to the diverse mathematical foundations and normalization approaches used in the MCDM techniques, reflecting how each method weighs and aggregates criteria differently. Regions such as Muvattupuzha (*R*_1_), Kothamangalam (*R*_5_), and Perumbavoor (*R*_10_) were consistently ranked toward the bottom, indicating lower vulnerability. These areas are either elevated or less urbanized, with natural vegetation cover that reduces surface runoff and helps mitigate flooding.

The efficiency of the proposed approach is evident in its ability to closely mirror real-time flood scenarios. The rankings generated by the hybrid LR-BTFN PSI–SAW–CODAS model correspond more realistically to the known flood patterns of Ernakulam district. In particular, the proposed method avoids rank inflation that occurs in classical crisp methods, since it effectively incorporates bipolar evaluations (positive and negative assessments) along with fuzziness through LR-type triangular numbers. This hybridization allows the model to handle both subjective expert judgments and objective variability in flood-related criteria, thereby yielding results that are not only mathematically robust but also practically reliable.

In summary, while conventional models provide reasonable rankings, the proposed method demonstrates efficient adaptability by aligning more closely with the ground-truth vulnerability of the study region. This confirms the practical applicability of the developed model for decision-makers in prioritizing flood-prone zones and planning targeted mitigation strategies.

#### 6.2.1 Classification and exploratory analysis of regions

**Figure 4:**
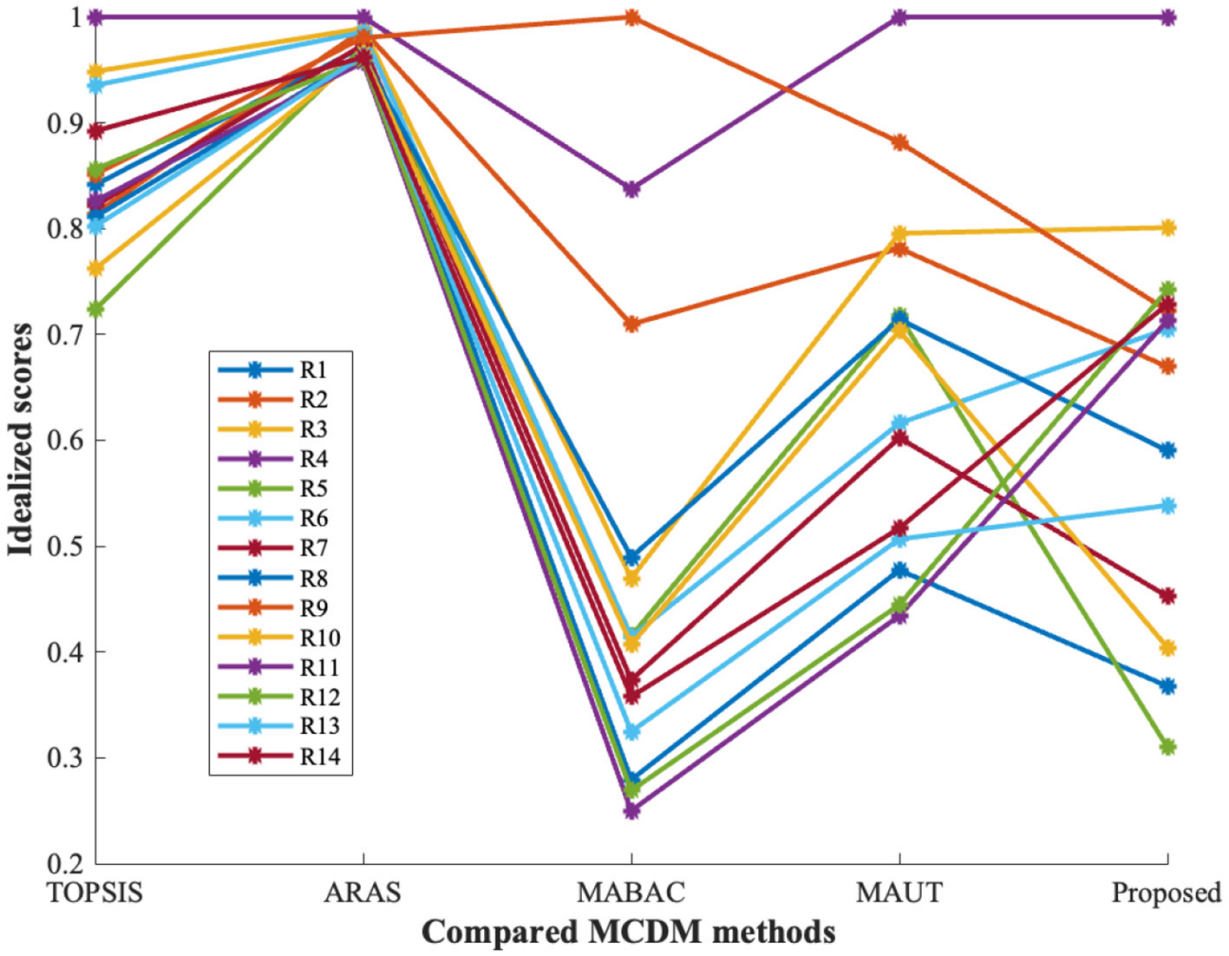
Comparative study of flood-prone zone prioritization.

**Figure 5:**
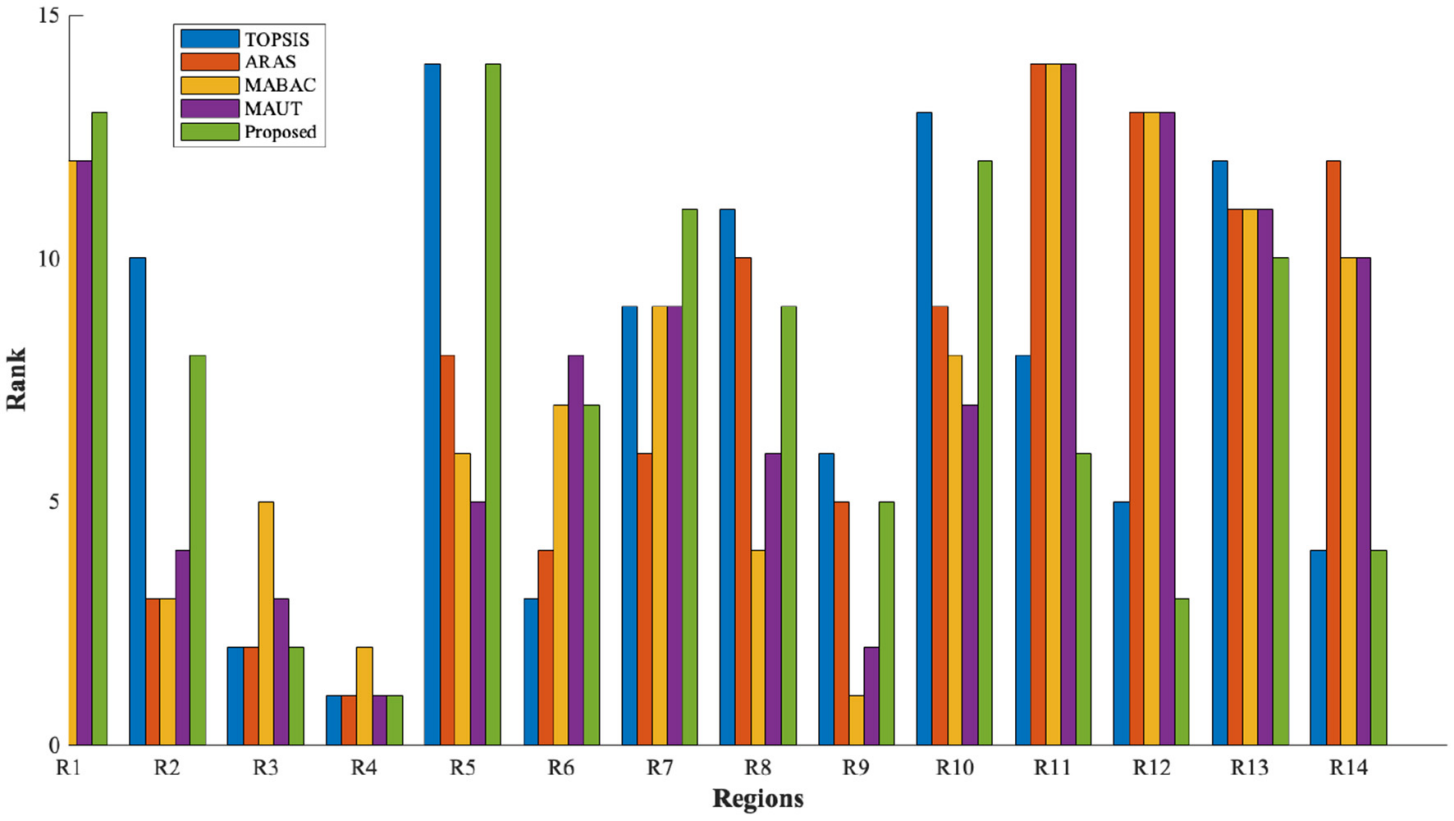
Flood vulnerability rankings (lower rank = higher priority).

**Figure 6:**
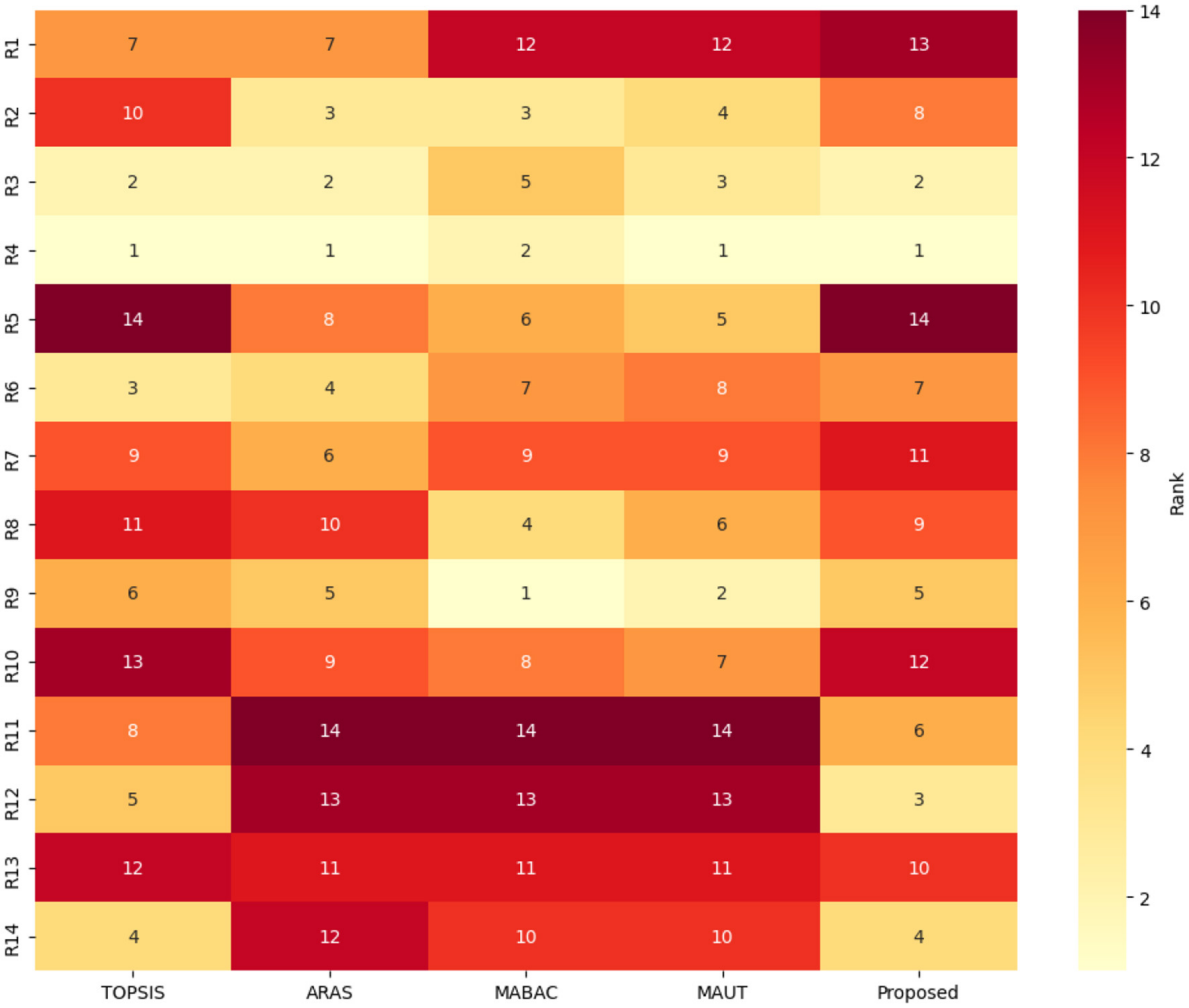
Heatmap of ranks across MCDM methods.

**Figure 7:**
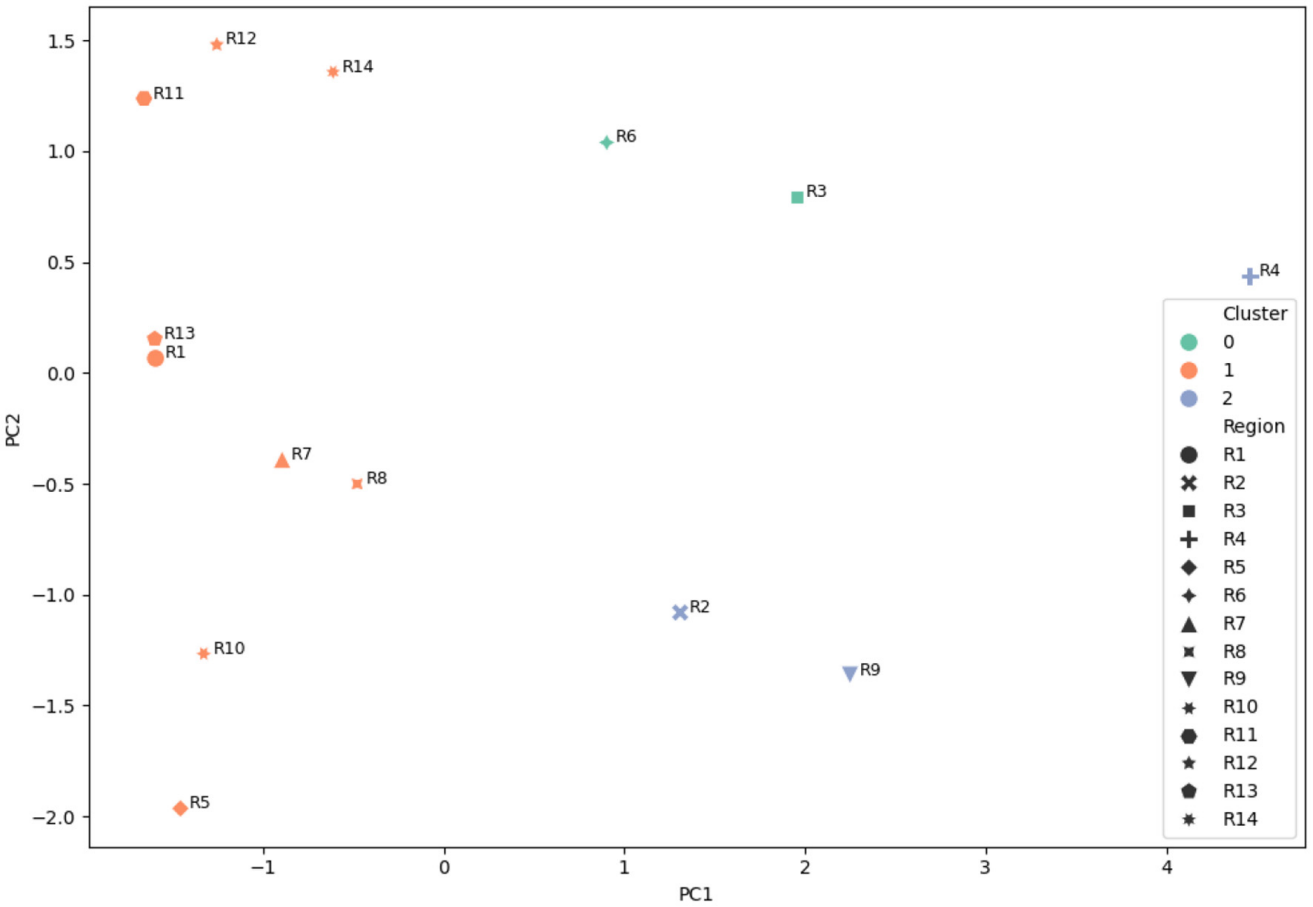
PCA of regions based on MCDM scores (clustered).

In addition to ranking the flood-prone regions, a classification analysis is performed to group regions with similar vulnerability characteristics. For this purpose, unsupervised learning techniques, namely K-means clustering and principal component analysis (PCA), are employed as exploratory tools to analyze patterns in the MCDM results. It is important to note that these methods are not used to validate the ranking accuracy of the proposed model. Instead, they provide supporting insights into the structural consistency and grouping behavior of the alternatives based on their vulnerability scores. From the observation of Figures 4–7, the following insights are obtained.

(i) Figure 4 illustrates the variation of scores across different MCDM methods. Regions such as *R*_4_, *R*_3_, and *R*_9_ consistently exhibit higher scores, indicating stable identification of highly vulnerable zones across methods.
(ii) Figure 5 presents the ranking distribution, highlighting agreement and variation among methods. Regions with consistent rankings indicate robust prioritization, whereas variations suggest sensitivity to methodological differences.
(iii) Figure 6 provides a visual representation of ranking patterns. Regions such as Kochi (*R*_4_) consistently appear in higher vulnerability zones across all methods, supporting the reliability of the ranking results.
(iv) K-means clustering groups the regions into three clusters based on similarity in vulnerability scores obtained from multiple MCDM methods. This grouping reflects natural divisions among low, moderate, and high flood-prone zones.

#### 6.2.2 Cluster characteristics

The clustering results categorize the regions into three groups:

(i) **Cluster 1 (Low Vulnerability):** Includes *R*_1_, *R*_5_, and *R*_10_, generally characterized by higher elevation and lower exposure to flood risk.
(ii) **Cluster 2 (Moderate Vulnerability):** Comprises *R*_2_, *R*_6_, *R*_7_, *R*_8_, *R*_11_, *R*_12_, *R*_13_, and *R*_14_, representing regions with mixed geographical and urban characteristics.
(iii) **Cluster 3 (High Vulnerability):** Includes *R*_3_, *R*_4_, and *R*_9_, which are predominantly coastal or river-adjacent regions with frequent flood exposure.

PCA is further used to project the multi-dimensional data into a two-dimensional space for visualization. The resulting plot (Figure 7) shows clear separation among the clusters, indicating that the vulnerability patterns derived from the MCDM models exhibit consistent structural behavior.

#### 6.2.3 Validation through real-world consistency and comparative analysis

While clustering and PCA provide useful exploratory insights, the primary validation of the proposed model is established through its consistency with real-world flood patterns and comparative performance. The high-ranking regions identified by the proposed model, such as Kochi, Vypen, and Paravoor, are well-documented flood-prone areas in the Ernakulam district, particularly during recent flood events. This alignment with observed flood occurrences supports the practical validity of the model. Furthermore, the comparative analysis with established MCDM methods demonstrates that the proposed approach yields stable and realistic rankings, with improved discrimination among moderately vulnerable regions.

Although more rigorous validation methods, such as correlation with historical flood datasets or predictive modeling, are beyond the scope of this study, they represent promising directions for future research.

### 6.3 Sensitivity analysis

To examine the robustness and reliability of the proposed hybrid PSI–SAW–CODAS model, a detailed sensitivity analysis was performed by introducing variations in both criteria weights and expert weights. Since the final ranking in MCDM models can be influenced by these parameters, it is essential to verify whether such variations significantly alter the decision outcomes.

The original ranking obtained from the proposed model identifies *R*_4_ as the most flood-prone region, followed by *R*_3_, *R*_12_, and *R*_14_, while *R*_5_ and *R*_1_ are ranked as the least vulnerable regions. This ranking is consistent with real-time flood observations in the Ernakulam district, where regions corresponding to higher ranks have historically experienced frequent flooding due to low elevation, proximity to water bodies, and high urbanization.

**(i) Sensitivity with respect to criteria weights:** In the first scenario, ten different sets of criteria weight vectors were generated as shown in Table A5 in Appendix A and applied to each expert decision matrix. As shown in Table A7 in Appendix A, the resulting rankings indicate that the top-ranked alternative (*R*_4_) consistently maintains its position across most cases, demonstrating strong stability. Similarly, the lowest-ranked alternatives (*R*_5_ and *R*_1_) also remain largely unchanged. Although minor fluctuations are observed among the middle-ranked alternatives (such as *R*_6_, *R*_11_, *R*_13_, and *R*_8_), these variations do not significantly impact the overall prioritization structure. Importantly, the group of highly vulnerable regions (top 4–5 alternatives) remains nearly identical across all cases, indicating that the model reliably identifies critical flood-prone zones even under varying criteria importance.

**(ii) Sensitivity with respect to expert weights:** In the second scenario, ten different combinations of expert weights were considered as shown in Table A6 in Appendix A to reflect variability in the influence of decision-makers. As given in Table A8 in Appendix A, the results show an even higher level of consistency compared to the criteria weight variation. The ranking order remains almost unchanged across all cases, with *R*_4_, *R*_3_, and *R*_12_ consistently appearing among the top-ranked alternatives, and *R*_5_ and *R*_1_ consistently appearing among the lowest-ranked ones. This stability indicates that the proposed model is not overly dependent on individual expert opinions and effectively integrates multiple perspectives without introducing significant bias.

**Impact of expert weight variations:**

It is observed that when the expert weights are varied randomly, certain changes in the ranking of alternatives occur. In particular, the alternative *R*_4_, which is ranked first in the proposed model, does not consistently retain its top position under arbitrary expert weight distributions. This behavior is expected, as the expert weights represent the relative importance and credibility of decision-makers in the aggregation process. When these weights are assigned randomly, without considering domain expertise, the resulting rankings may reflect biased or less reliable evaluations. However, under the original expert weight configuration, which is based on the expertise and experience of the selected domain experts, the model consistently identifies *R*_4_ as the most vulnerable region. This outcome is also supported by historical flood records in the study area. Therefore, the sensitivity analysis indicates that while the model is robust with respect to variations in criteria weights, the ranking outcomes are influenced by the assignment of expert weights, emphasizing the importance of informed and justified expert selection in group decision-making problems.

The sensitivity analysis clearly demonstrates that the proposed model exhibits strong robustness against variations in both criteria and expert weights. The consistency of top-ranked alternatives is particularly significant, as these regions correspond to known flood-prone zones in the real-world context of the Ernakulam district. Moreover, the model preserves the relative ordering of alternatives with high reliability, while only allowing minor and expected variations among closely ranked regions. This behavior reflects a desirable characteristic of decision-making models, where critical decisions (such as identifying high-risk zones) remain stable, even under uncertain conditions. Overall, the results confirm that the proposed hybrid PSI–SAW–CODAS model provides stable, reliable, and practically consistent rankings, reinforcing its suitability for real-world flood hazard assessment and decision support.

### 6.4 Policy implications

This comparative study provides evidence-based insights that can inform flood mitigation planning in Ernakulam:

(i) High-priority zones such as Kochi (*R*_4_), Vypen (*R*_3_), and Aluva (*R*_9_) should be targeted for urgent interventions, including flood barriers, improved drainage, and early warning systems.
(ii) Moderate-risk areas can benefit from zoning regulations, improved green cover, and community preparedness programs.
(iii) Low-risk regions should maintain natural flood buffers and monitor potential risks due to urbanization.

The combined application of MCDM enables a multi-perspective, robust analysis that aids decision-makers in allocating resources and designing effective flood management strategies.

This study utilized five MCDM methods to evaluate and prioritize flood vulnerability across 14 regions in the Ernakulam district. The results demonstrate that while traditional models like TOPSIS and ARAS are effective in identifying extreme cases, the proposed fuzzy hybrid model offers improved interpretability and reliability, especially in mid-priority zones.

By incorporating validation through ML techniques, such as clustering and PCA, the study goes beyond simple ranking to uncover meaningful regional groupings and trends. This integrated approach can serve as a valuable tool for urban planners, disaster management authorities, and local governments aiming to implement data-driven flood risk mitigation strategies.

### 6.5 Limitations of the work

The limitations of the present work are discussed as follows:

(i) The framework incorporates linguistic assessments from a limited group of domain experts; although fuzzy modeling reduces uncertainty, some degree of subjectivity remains unavoidable.
(ii) The effectiveness of the proposed approach is demonstrated using data from Ernakulam district, and its application to other geographical regions may require contextual adaptation of criteria and inputs.
(iii) The current analysis is conducted using static datasets and does not explicitly account for temporal dynamics or real-time flood progression and climate variability.
(iv) The absence of large-scale labeled datasets for the study region further limits the applicability of supervised machine learning models in the present context.

## 7 Conclusion

The findings of this study demonstrate that the hybrid PSI–SAW–CODAS framework under the LRBTFN setting offers a reliable and context-aware approach for assessing regional flood vulnerability. By integrating expert linguistic inputs with key hydrological, environmental, and socio-geographical criteria, the model successfully ranked 14 regions in Ernakulam with a high degree of realism and consistency. Areas such as Kochi, Vypen, and Paravoor emerged as the most flood-sensitive, reflecting their coastal exposure, historical flood records, and rapid urbanization, whereas Kothamangalam showed minimal susceptibility. The comparative assessment with TOPSIS, ARAS, MABAC, and MAUT highlighted that the proposed hybrid approach delivers rankings that correspond more closely with real flood events and official disaster records. Unlike conventional crisp methods, the LRBTFN-based framework effectively handled uncertainty, bipolar assessments and subjective–objective integration, thereby minimizing rank distortion and enhancing decision reliability. Visualization tools such as line graphs, bar charts and heatmaps, along with unsupervised methods like K-Means clustering and PCA, further reinforced the consistency of regional vulnerability patterns. Moreover, the proposed approach provides policymakers, urban planners and disaster management authorities with a robust and adaptable decision-support tool for mitigating flood risks and enhancing resilience.

Future work may extend this model (i) by incorporating GIS-based spatial data, temporal flood records, and climate change projections to capture dynamic environmental variations and strengthen predictive capability. (ii) by exploring hybrid models that integrate data-driven predictive capabilities with fuzzy decision-making frameworks to enhance both accuracy and interpretability in flood risk assessment. (iii) by integrating methods such as DEMATEL, AHP, or ANP to explicitly model interdependencies among criteria.

## Data Availability

The original contributions presented in the study are included in the article/Supplementary material, further inquiries can be directed to the corresponding author.
