# Supplementary material for: Flood hazard assessment and zonal prioritization through an LR-bipolar triangular fuzzy hybrid decision-making approach
Source: Front Artif Intell. 2026 May 1;9:1817216. doi: 10.3389/frai.2026.1817216 (PMC13176306; doi:10.3389/frai.2026.1817216)
Supplement: Supplementary file 1 [file Table_1.pdf]

Flood Hazard Assessment and Zonal Prioritization through an LR-Bipolar Triangular Fuzzy Hybrid Decision-Making Approach

A Appendix: Tabulated results of the computations using the proposed model

The decision matrices with respect to four experts, 10 cases of criteria weights with respect to each decision matrix and expert weights for sensitivity analysis are tabulated in the Appendix A.

Table A1: LR-bipolar triangular fuzzy decision matrix with respect to Expert 1

	C_1	C_2	C_3	C_4	C_5
A_1	((0.5,0.1,0.1),(0.5,0.1,0.1))	((0.8,0.1,0.05),(0.2,0.05,0.1))	((0.7,0.05,0.1),(0.3,0.1,0.05))	((0.5,0.1,0.1),(0.5,0.1,0.1))	((0.8,0.1,0.05),(0.2,0.05,0.1))
A_2	((0.3,0.1,0.05),(0.7,0.05,0.1))	((0.95,0.1,0),(0.05,0,0.1))	((0.7,0.05,0.1),(0.3,0.1,0.05))	((0.2,0.1,0.05),(0.8,0.05,0.1))	((0.7,0.05,0.1),(0.3,0.1,0.05))
A_3	((0.7,0.05,0.1),(0.3,0.1,0.05))	((0.8,0.1,0.05),(0.2,0.05,0.1))	((0.8,0.1,0.05),(0.2,0.05,0.1))	((0.3,0.1,0.05),(0.7,0.05,0.1))	((0.8,0.1,0.05),(0.2,0.05,0.1))
A_4	((0.2,0.1,0.05),(0.8,0.05,0.1))	((0.9,0.05,0.1),(0.1,0.05,0.05))	((0.9,0.05,0.1),(0.1,0.05,0.05))	((0.2,0.1,0.05),(0.8,0.05,0.1))	((0.9,0.05,0.1),(0.1,0.05,0.05))
A_5	((0.2,0.1,0.05),(0.8,0.05,0.1))	((0.7,0.05,0.1),(0.3,0.1,0.05))	((0.8,0.1,0.05),(0.2,0.05,0.1))	((0.8,0.1,0.05),(0.2,0.05,0.1))	((0.8,0.1,0.05),(0.2,0.05,0.1))
A_6	((0.1,0.05,0.1),(0.9,0.1,0.05))	((0.8,0.1,0.05),(0.2,0.05,0.1))	((0.9,0.05,0.1),(0.1,0.05,0.05))	((0.2,0.1,0.05),(0.8,0.05,0.1))	((0.9,0.05,0.1),(0.1,0.05,0.05))
A_7	((0.5,0.1,0.1),(0.5,0.1,0.1))	((0.8,0.1,0.05),(0.2,0.05,0.1))	((0.9,0.05,0.1),(0.1,0.05,0.05))	((0.5,0.1,0.1),(0.5,0.1,0.1))	((0.9,0.05,0.1),(0.1,0.05,0.05))
A_8	((0.8,0.1,0.05),(0.2,0.05,0.1))	((0.7,0.05,0.1),(0.3,0.1,0.05))	((0.8,0.1,0.05),(0.2,0.05,0.1))	((0.5,0.1,0.1),(0.5,0.1,0.1))	((0.8,0.1,0.05),(0.2,0.05,0.1))
A_9	((0.5,0.1,0.1),(0.5,0.1,0.1))	((0.95,0.1,0),(0.05,0,0.1))	((0.9,0.05,0.1),(0.1,0.05,0.05))	((0.5,0.1,0.1),(0.5,0.1,0.1))	((0.8,0.1,0.05),(0.2,0.05,0.1))
A_{10}	((0.7,0.05,0.1),(0.3,0.1,0.05))	((0.7,0.05,0.1),(0.3,0.1,0.05))	((0.8,0.1,0.05),(0.2,0.05,0.1))	((0.5,0.1,0.1),(0.5,0.1,0.1))	((0.7,0.05,0.1),(0.3,0.1,0.05))
A_{11}	((0.8,0.1,0.05),(0.2,0.05,0.1))	((0.5,0.1,0.1),(0.5,0.1,0.1))	((0.7,0.05,0.1),(0.3,0.1,0.05))	((0.8,0.1,0.05),(0.2,0.05,0.1))	((0.5,0.1,0.1),(0.5,0.1,0.1))
A_{12}	((0.8,0.1,0.05),(0.2,0.05,0.1))	((0.5,0.1,0.1),(0.5,0.1,0.1))	((0.7,0.05,0.1),(0.3,0.1,0.05))	((0.8,0.1,0.05),(0.2,0.05,0.1))	((0.5,0.1,0.1),(0.5,0.1,0.1))
A_{13}	((0.8,0.1,0.05),(0.2,0.05,0.1))	((0.3,0.1,0.05),(0.7,0.05,0.1))	((0.5,0.1,0.1),(0.5,0.1,0.1))	((0.7,0.05,0.1),(0.3,0.1,0.05))	((0.8,0.1,0.05),(0.2,0.05,0.1))
A_{14}	((0.9,0.05,0.1),(0.1,0.05,0.05))	((0.3,0.1,0.05),(0.7,0.05,0.1))	((0.5,0.1,0.1),(0.5,0.1,0.1))	((0.8,0.1,0.05),(0.2,0.05,0.1))	((0.5,0.1,0.1),(0.5,0.1,0.1))

	C_6	C_7	C_8	C_9	C_{10}
A_1	((0.5,0.1,0.1),(0.5,0.1,0.1))	((0.8,0.1,0.05),(0.2,0.05,0.1))	((0.5,0.1,0.1),(0.5,0.1,0.1))	((0.9,0.05,0.1),(0.1,0.05,0.05))	((0.5,0.1,0.1),(0.5,0.1,0.1))
A_2	((0.7,0.05,0.1),(0.3,0.1,0.05))	((0.8,0.1,0.05),(0.2,0.05,0.1))	((0.9,0.05,0.1),(0.1,0.05,0.05))	((0.8,0.1,0.05),(0.2,0.05,0.1))	((0.8,0.1,0.05),(0.2,0.05,0.1))
A_3	((0.5,0.1,0.1),(0.5,0.1,0.1))	((0.7,0.05,0.1),(0.3,0.1,0.05))	((0.9,0.05,0.1),(0.1,0.05,0.05))	((0.8,0.1,0.05),(0.2,0.05,0.1))	((0.7,0.05,0.1),(0.3,0.1,0.05))
A_4	((0.2,0.1,0.05),(0.8,0.05,0.1))	((0.8,0.1,0.05),(0.2,0.05,0.1))	((0.9,0.05,0.1),(0.1,0.05,0.05))	((0.9,0.05,0.1),(0.1,0.05,0.05))	((0.2,0.1,0.05),(0.8,0.05,0.1))
A_5	((0.5,0.1,0.1),(0.5,0.1,0.1))	((0.8,0.1,0.05),(0.2,0.05,0.1))	((0.7,0.05,0.1),(0.3,0.1,0.05))	((0.8,0.1,0.05),(0.2,0.05,0.1))	((0.5,0.1,0.1),(0.5,0.1,0.1))
A_6	((0.5,0.1,0.1),(0.5,0.1,0.1))	((0.9,0.05,0.1),(0.1,0.05,0.05))	((0.9,0.05,0.1),(0.1,0.05,0.05))	((0.9,0.05,0.1),(0.1,0.05,0.05))	((0.2,0.1,0.05),(0.8,0.05,0.1))
A_7	((0.7,0.05,0.1),(0.3,0.1,0.05))	((0.7,0.05,0.1),(0.3,0.1,0.05))	((0.7,0.05,0.1),(0.3,0.1,0.05))	((0.8,0.1,0.05),(0.2,0.05,0.1))	((0.5,0.1,0.1),(0.5,0.1,0.1))
A_8	((0.8,0.1,0.05),(0.2,0.05,0.1))	((0.9,0.05,0.1),(0.1,0.05,0.05))	((0.2,0.1,0.05),(0.8,0.05,0.1))	((0.8,0.1,0.05),(0.2,0.05,0.1))	((0.8,0.1,0.05),(0.2,0.05,0.1))
A_9	((0.7,0.05,0.1),(0.3,0.1,0.05))	((0.9,0.05,0.1),(0.1,0.05,0.05))	((0.9,0.05,0.1),(0.1,0.05,0.05))	((0.9,0.05,0.1),(0.1,0.05,0.05))	((0.5,0.1,0.1),(0.5,0.1,0.1))
A_{10}	((0.8,0.1,0.05),(0.2,0.05,0.1))	((0.7,0.05,0.1),(0.3,0.1,0.05))	((0.7,0.05,0.1),(0.3,0.1,0.05))	((0.5,0.1,0.1),(0.5,0.1,0.1))	((0.5,0.1,0.1),(0.5,0.1,0.1))
A_{11}	((0.7,0.05,0.1),(0.3,0.1,0.05))	((0.8,0.1,0.05),(0.2,0.05,0.1))	((0.5,0.1,0.1),(0.5,0.1,0.1))	((0.5,0.1,0.1),(0.5,0.1,0.1))	((0.8,0.1,0.05),(0.2,0.05,0.1))
A_{12}	((0.7,0.05,0.1),(0.3,0.1,0.05))	((0.5,0.1,0.1),(0.5,0.1,0.1))	((0.7,0.05,0.1),(0.3,0.1,0.05))	((0.3,0.1,0.05),(0.7,0.05,0.1))	((0.9,0.05,0.1),(0.1,0.05,0.05))
A_{13}	((0.5,0.1,0.1),(0.5,0.1,0.1))	((0.7,0.05,0.1),(0.3,0.1,0.05))	((0.8,0.1,0.05),(0.2,0.05,0.1))	((0.5,0.1,0.1),(0.5,0.1,0.1))	((0.8,0.1,0.05),(0.2,0.05,0.1))
A_{14}	((0.3,0.1,0.05),(0.7,0.05,0.1))	((0.5,0.1,0.1),(0.5,0.1,0.1))	((0.5,0.1,0.1),(0.5,0.1,0.1))	((0.3,0.1,0.05),(0.7,0.05,0.1))	((0.9,0.05,0.1),(0.1,0.05,0.05))

Table A2: LR-bipolar triangular fuzzy decision matrix with respect to Expert 2

	C_1	C_2	C_3	C_4	C_5
A_1	((0.7,0.05,0.1),(0.3,0.1,0.05))	((0.5,0.1,0.1),(0.5,0.1,0.1))	((0.5,0.1,0.1),(0.5,0.1,0.1))	((0.8,0.1,0.05),(0.2,0.05,0.1))	((0.5,0.1,0.1),(0.5,0.1,0.1))
A_2	((0.1,0.05,0.1),(0.9,0.1,0.05))	((0.95,0.1,0),(0.05,0,0.1))	((0.5,0.1,0.1),(0.5,0.1,0.1))	((0.2,0.1,0.05),(0.8,0.05,0.1))	((0.5,0.1,0.1),(0.5,0.1,0.1))
A_3	((0.05,0,0.1),(0.95,0.05,0))	((0.9,0.05,0.1),(0.1,0.05,0.05))	((0.5,0.1,0.1),(0.5,0.1,0.1))	((0.05,0,0.1),(0.95,0.05,0))	((0.2,0.1,0.05),(0.8,0.05,0.1))
A_4	((0.05,0,0.1),(0.95,0.05,0))	((0.8,0.1,0.05),(0.2,0.05,0.1))	((0.5,0.1,0.1),(0.5,0.1,0.1))	((0.2,0.1,0.05),(0.8,0.05,0.1))	((0.9,0.05,0.1),(0.1,0.05,0.05))
A_5	((0.3,0.1,0.05),(0.7,0.05,0.1))	((0.5,0.1,0.1),(0.5,0.1,0.1))	((0.7,0.05,0.1),(0.3,0.1,0.05))	((0.5,0.1,0.1),(0.5,0.1,0.1))	((0.5,0.1,0.1),(0.5,0.1,0.1))
A_6	((0.2,0.1,0.05),(0.8,0.05,0.1))	((0.8,0.1,0.05),(0.2,0.05,0.1))	((0.3,0.1,0.05),(0.7,0.05,0.1))	((0.2,0.1,0.05),(0.8,0.05,0.1))	((0.8,0.1,0.05),(0.2,0.05,0.1))
A_7	((0.3,0.1,0.05),(0.7,0.05,0.1))	((0.2,0.1,0.05),(0.8,0.05,0.1))	((0.5,0.1,0.1),(0.5,0.1,0.1))	((0.8,0.1,0.05),(0.2,0.05,0.1))	((0.5,0.1,0.1),(0.5,0.1,0.1))
A_8	((0.5,0.1,0.1),(0.5,0.1,0.1))	((0.2,0.1,0.05),(0.8,0.05,0.1))	((0.7,0.05,0.1),(0.3,0.1,0.05))	((0.7,0.05,0.1),(0.3,0.1,0.05))	((0.5,0.1,0.1),(0.5,0.1,0.1))
A_9	((0.2,0.1,0.05),(0.8,0.05,0.1))	((0.8,0.1,0.05),(0.2,0.05,0.1))	((0.5,0.1,0.1),(0.5,0.1,0.1))	((0.3,0.1,0.05),(0.7,0.05,0.1))	((0.5,0.1,0.1),(0.5,0.1,0.1))
A_{10}	((0.5,0.1,0.1),(0.5,0.1,0.1))	((0.2,0.1,0.05),(0.8,0.05,0.1))	((0.7,0.05,0.1),(0.3,0.1,0.05))	((0.5,0.1,0.1),(0.5,0.1,0.1))	((0.5,0.1,0.1),(0.5,0.1,0.1))
A_{11}	((0.5,0.1,0.1),(0.5,0.1,0.1))	((0.2,0.1,0.05),(0.8,0.05,0.1))	((0.5,0.1,0.1),(0.5,0.1,0.1))	((0.7,0.05,0.1),(0.3,0.1,0.05))	((0.5,0.1,0.1),(0.5,0.1,0.1))
A_{12}	((0.5,0.1,0.1),(0.5,0.1,0.1))	((0.2,0.1,0.05),(0.8,0.05,0.1))	((0.5,0.1,0.1),(0.5,0.1,0.1))	((0.5,0.1,0.1),(0.5,0.1,0.1))	((0.5,0.1,0.1),(0.5,0.1,0.1))
A_{13}	((0.7,0.05,0.1),(0.3,0.1,0.05))	((0.2,0.1,0.05),(0.8,0.05,0.1))	((0.7,0.05,0.1),(0.3,0.1,0.05))	((0.7,0.05,0.1),(0.3,0.1,0.05))	((0.5,0.1,0.1),(0.5,0.1,0.1))
A_{14}	((0.8,0.1,0.05),(0.2,0.05,0.1))	((0.2,0.1,0.05),(0.8,0.05,0.1))	((0.8,0.1,0.05),(0.2,0.05,0.1))	((0.5,0.1,0.1),(0.5,0.1,0.1))	((0.5,0.1,0.1),(0.5,0.1,0.1))

	C_6	C_7	C_8	C_9	C_{10}
A_1	((0.8,0.1,0.05),(0.2,0.05,0.1))	((0.5,0.1,0.1),(0.5,0.1,0.1))	((0.5,0.1,0.1),(0.5,0.1,0.1))	((0.5,0.1,0.1),(0.5,0.1,0.1))	((0.5,0.1,0.1),(0.5,0.1,0.1))
A_2	((0.2,0.1,0.05),(0.8,0.05,0.1))	((0.2,0.1,0.05),(0.8,0.05,0.1))	((0.8,0.1,0.05),(0.2,0.05,0.1))	((0.5,0.1,0.1),(0.5,0.1,0.1))	((0.5,0.1,0.1),(0.5,0.1,0.1))
A_3	((0.2,0.1,0.05),(0.8,0.05,0.1))	((0.2,0.1,0.05),(0.8,0.05,0.1))	((0.9,0.05,0.1),(0.1,0.05,0.05))	((0.9,0.05,0.1),(0.1,0.05,0.05))	((0.5,0.1,0.1),(0.5,0.1,0.1))
A_4	((0.2,0.1,0.05),(0.8,0.05,0.1))	((0.9,0.05,0.1),(0.1,0.05,0.05))	((0.5,0.1,0.1),(0.5,0.1,0.1))	((0.9,0.05,0.1),(0.1,0.05,0.05))	((0.5,0.1,0.1),(0.5,0.1,0.1))
A_5	((0.5,0.1,0.1),(0.5,0.1,0.1))	((0.7,0.05,0.1),(0.3,0.1,0.05))	((0.5,0.1,0.1),(0.5,0.1,0.1))	((0.5,0.1,0.1),(0.5,0.1,0.1))	((0.7,0.05,0.1),(0.3,0.1,0.05))
A_6	((0.5,0.1,0.1),(0.5,0.1,0.1))	((0.8,0.1,0.05),(0.2,0.05,0.1))	((0.5,0.1,0.1),(0.5,0.1,0.1))	((0.8,0.1,0.05),(0.2,0.05,0.1))	((0.5,0.1,0.1),(0.5,0.1,0.1))
A_7	((0.5,0.1,0.1),(0.5,0.1,0.1))	((0.7,0.05,0.1),(0.3,0.1,0.05))	((0.5,0.1,0.1),(0.5,0.1,0.1))	((0.5,0.1,0.1),(0.5,0.1,0.1))	((0.5,0.1,0.1),(0.5,0.1,0.1))
A_8	((0.5,0.1,0.1),(0.5,0.1,0.1))	((0.7,0.05,0.1),(0.3,0.1,0.05))	((0.7,0.05,0.1),(0.3,0.1,0.05))	((0.5,0.1,0.1),(0.5,0.1,0.1))	((0.7,0.05,0.1),(0.3,0.1,0.05))
A_9	((0.9,0.05,0.1),(0.1,0.05,0.05))	((0.8,0.1,0.05),(0.2,0.05,0.1))	((0.5,0.1,0.1),(0.5,0.1,0.1))	((0.8,0.1,0.05),(0.2,0.05,0.1))	((0.5,0.1,0.1),(0.5,0.1,0.1))
A_{10}	((0.5,0.1,0.1),(0.5,0.1,0.1))	((0.7,0.05,0.1),(0.3,0.1,0.05))	((0.5,0.1,0.1),(0.5,0.1,0.1))	((0.5,0.1,0.1),(0.5,0.1,0.1))	((0.7,0.05,0.1),(0.3,0.1,0.05))
A_{11}	((0.5,0.1,0.1),(0.5,0.1,0.1))	((0.5,0.1,0.1),(0.5,0.1,0.1))	((0.5,0.1,0.1),(0.5,0.1,0.1))	((0.5,0.1,0.1),(0.5,0.1,0.1))	((0.5,0.1,0.1),(0.5,0.1,0.1))
A_{12}	((0.5,0.1,0.1),(0.5,0.1,0.1))	((0.5,0.1,0.1),(0.5,0.1,0.1))	((0.5,0.1,0.1),(0.5,0.1,0.1))	((0.5,0.1,0.1),(0.5,0.1,0.1))	((0.5,0.1,0.1),(0.5,0.1,0.1))
A_{13}	((0.8,0.1,0.05),(0.2,0.05,0.1))	((0.5,0.1,0.1),(0.5,0.1,0.1))	((0.8,0.1,0.05),(0.2,0.05,0.1))	((0.5,0.1,0.1),(0.5,0.1,0.1))	((0.8,0.1,0.05),(0.2,0.05,0.1))
A_{14}	((0.5,0.1,0.1),(0.5,0.1,0.1))	((0.5,0.1,0.1),(0.5,0.1,0.1))	((0.8,0.1,0.05),(0.2,0.05,0.1))	((0.5,0.1,0.1),(0.5,0.1,0.1))	((0.8,0.1,0.05),(0.2,0.05,0.1))

Table A3: LR-bipolar triangular fuzzy decision matrix with respect to Expert 3

	C_1	C_2	C_3	C_4	C_5
A_1	((0.7,0.05,0.1),(0.3,0.1,0.05))	((0.5,0.1,0.1),(0.5,0.1,0.1))	((0.5,0.1,0.1),(0.5,0.1,0.1))	((0.5,0.1,0.1),(0.5,0.1,0.1))	((0.8,0.1,0.05),(0.2,0.05,0.1))
A_2	((0.1,0.05,0.1),(0.9,0.1,0.05))	((0.95,0.1,0),(0.05,0,0.1))	((0.5,0.1,0.1),(0.5,0.1,0.1))	((0.2,0.1,0.05),(0.8,0.05,0.1))	((0.5,0.1,0.1),(0.5,0.1,0.1))
A_3	((0.05,0,0.1),(0.95,0.05,0))	((0.9,0.05,0.1),(0.1,0.05,0.05))	((0.5,0.1,0.1),(0.5,0.1,0.1))	((0.05,0,0.1),(0.95,0.05,0))	((0.2,0.1,0.05),(0.8,0.05,0.1))
A_4	((0.05,0,0.1),(0.95,0.05,0))	((0.8,0.1,0.05),(0.2,0.05,0.1))	((0.5,0.1,0.1),(0.5,0.1,0.1))	((0.2,0.1,0.05),(0.8,0.05,0.1))	((0.9,0.05,0.1),(0.1,0.05,0.05))
A_5	((0.3,0.1,0.05),(0.7,0.05,0.1))	((0.5,0.1,0.1),(0.5,0.1,0.1))	((0.5,0.1,0.1),(0.5,0.1,0.1))	((0.7,0.05,0.1),(0.3,0.1,0.05))	((0.5,0.1,0.1),(0.5,0.1,0.1))
A_6	((0.2,0.1,0.05),(0.8,0.05,0.1))	((0.8,0.1,0.05),(0.2,0.05,0.1))	((0.3,0.1,0.05),(0.7,0.05,0.1))	((0.2,0.1,0.05),(0.8,0.05,0.1))	((0.8,0.1,0.05),(0.2,0.05,0.1))
A_7	((0.3,0.1,0.05),(0.7,0.05,0.1))	((0.2,0.1,0.05),(0.8,0.05,0.1))	((0.5,0.1,0.1),(0.5,0.1,0.1))	((0.5,0.1,0.1),(0.5,0.1,0.1))	((0.8,0.1,0.05),(0.2,0.05,0.1))
A_8	((0.5,0.1,0.1),(0.5,0.1,0.1))	((0.2,0.1,0.05),(0.8,0.05,0.1))	((0.7,0.05,0.1),(0.3,0.1,0.05))	((0.7,0.05,0.1),(0.3,0.1,0.05))	((0.5,0.1,0.1),(0.5,0.1,0.1))
A_9	((0.2,0.1,0.05),(0.8,0.05,0.1))	((0.8,0.1,0.05),(0.2,0.05,0.1))	((0.5,0.1,0.1),(0.5,0.1,0.1))	((0.5,0.1,0.1),(0.5,0.1,0.1))	((0.3,0.1,0.05),(0.7,0.05,0.1))
A_{10}	((0.5,0.1,0.1),(0.5,0.1,0.1))	((0.2,0.1,0.05),(0.8,0.05,0.1))	((0.7,0.05,0.1),(0.3,0.1,0.05))	((0.7,0.05,0.1),(0.3,0.1,0.05))	((0.5,0.1,0.1),(0.5,0.1,0.1))
A_{11}	((0.5,0.1,0.1),(0.5,0.1,0.1))	((0.2,0.1,0.05),(0.8,0.05,0.1))	((0.5,0.1,0.1),(0.5,0.1,0.1))	((0.7,0.05,0.1),(0.3,0.1,0.05))	((0.5,0.1,0.1),(0.5,0.1,0.1))
A_{12}	((0.5,0.1,0.1),(0.5,0.1,0.1))	((0.2,0.1,0.05),(0.8,0.05,0.1))	((0.5,0.1,0.1),(0.5,0.1,0.1))	((0.5,0.1,0.1),(0.5,0.1,0.1))	((0.5,0.1,0.1),(0.5,0.1,0.1))
A_{13}	((0.7,0.05,0.1),(0.3,0.1,0.05))	((0.2,0.1,0.05),(0.8,0.05,0.1))	((0.7,0.05,0.1),(0.3,0.1,0.05))	((0.7,0.05,0.1),(0.3,0.1,0.05))	((0.5,0.1,0.1),(0.5,0.1,0.1))
A_{14}	((0.8,0.1,0.05),(0.2,0.05,0.1))	((0.2,0.1,0.05),(0.8,0.05,0.1))	((0.8,0.1,0.05),(0.2,0.05,0.1))	((0.8,0.1,0.05),(0.2,0.05,0.1))	((0.5,0.1,0.1),(0.5,0.1,0.1))

	C_6	C_7	C_8	C_9	C_{10}
A_1	((0.5,0.1,0.1),(0.5,0.1,0.1))	((0.8,0.1,0.05),(0.2,0.05,0.1))	((0.5,0.1,0.1),(0.5,0.1,0.1))	((0.5,0.1,0.1),(0.5,0.1,0.1))	((0.5,0.1,0.1),(0.5,0.1,0.1))
A_2	((0.2,0.1,0.05),(0.8,0.05,0.1))	((0.2,0.1,0.05),(0.8,0.05,0.1))	((0.2,0.1,0.05),(0.8,0.05,0.1))	((0.8,0.1,0.05),(0.2,0.05,0.1))	((0.5,0.1,0.1),(0.5,0.1,0.1))
A_3	((0.2,0.1,0.05),(0.8,0.05,0.1))	((0.2,0.1,0.05),(0.8,0.05,0.1))	((0.2,0.1,0.05),(0.8,0.05,0.1))	((0.9,0.05,0.1),(0.1,0.05,0.05))	((0.9,0.05,0.1),(0.1,0.05,0.05))
A_4	((0.2,0.1,0.05),(0.8,0.05,0.1))	((0.5,0.1,0.1),(0.5,0.1,0.1))	((0.5,0.1,0.1),(0.5,0.1,0.1))	((0.9,0.05,0.1),(0.1,0.05,0.05))	((0.9,0.05,0.1),(0.1,0.05,0.05))
A_5	((0.5,0.1,0.1),(0.5,0.1,0.1))	((0.5,0.1,0.1),(0.5,0.1,0.1))	((0.5,0.1,0.1),(0.5,0.1,0.1))	((0.5,0.1,0.1),(0.5,0.1,0.1))	((0.7,0.05,0.1),(0.3,0.1,0.05))
A_6	((0.2,0.1,0.05),(0.8,0.05,0.1))	((0.5,0.1,0.1),(0.5,0.1,0.1))	((0.5,0.1,0.1),(0.5,0.1,0.1))	((0.8,0.1,0.05),(0.2,0.05,0.1))	((0.8,0.1,0.05),(0.2,0.05,0.1))
A_7	((0.5,0.1,0.1),(0.5,0.1,0.1))	((0.5,0.1,0.1),(0.5,0.1,0.1))	((0.5,0.1,0.1),(0.5,0.1,0.1))	((0.5,0.1,0.1),(0.5,0.1,0.1))	((0.7,0.05,0.1),(0.3,0.1,0.05))
A_8	((0.7,0.05,0.1),(0.3,0.1,0.05))	((0.5,0.1,0.1),(0.5,0.1,0.1))	((0.5,0.1,0.1),(0.5,0.1,0.1))	((0.5,0.1,0.1),(0.5,0.1,0.1))	((0.7,0.05,0.1),(0.3,0.1,0.05))
A_9	((0.5,0.1,0.1),(0.5,0.1,0.1))	((0.5,0.1,0.1),(0.5,0.1,0.1))	((0.9,0.05,0.1),(0.1,0.05,0.05))	((0.8,0.1,0.05),(0.2,0.05,0.1))	((0.5,0.1,0.1),(0.5,0.1,0.1))
A_{10}	((0.5,0.1,0.1),(0.5,0.1,0.1))	((0.5,0.1,0.1),(0.5,0.1,0.1))	((0.5,0.1,0.1),(0.5,0.1,0.1))	((0.5,0.1,0.1),(0.5,0.1,0.1))	((0.7,0.05,0.1),(0.3,0.1,0.05))
A_{11}	((0.5,0.1,0.1),(0.5,0.1,0.1))	((0.5,0.1,0.1),(0.5,0.1,0.1))	((0.5,0.1,0.1),(0.5,0.1,0.1))	((0.5,0.1,0.1),(0.5,0.1,0.1))	((0.5,0.1,0.1),(0.5,0.1,0.1))
A_{12}	((0.5,0.1,0.1),(0.5,0.1,0.1))	((0.5,0.1,0.1),(0.5,0.1,0.1))	((0.5,0.1,0.1),(0.5,0.1,0.1))	((0.5,0.1,0.1),(0.5,0.1,0.1))	((0.5,0.1,0.1),(0.5,0.1,0.1))
A_{13}	((0.7,0.05,0.1),(0.3,0.1,0.05))	((0.5,0.1,0.1),(0.5,0.1,0.1))	((0.8,0.1,0.05),(0.2,0.05,0.1))	((0.5,0.1,0.1),(0.5,0.1,0.1))	((0.8,0.1,0.05),(0.2,0.05,0.1))
A_{14}	((0.8,0.1,0.05),(0.2,0.05,0.1))	((0.5,0.1,0.1),(0.5,0.1,0.1))	((0.5,0.1,0.1),(0.5,0.1,0.1))	((0.5,0.1,0.1),(0.5,0.1,0.1))	((0.8,0.1,0.05),(0.2,0.05,0.1))

Table A4: LR-bipolar triangular fuzzy decision matrix with respect to Expert 4

	C_1	C_2	C_3	C_4	C_5
A_1	((0.3,0.1,0.05),(0.7,0.05,0.1))	((0.2,0.1,0.05),(0.8,0.05,0.1))	((0.1,0.05,0.1),(0.9,0.1,0.05))	((0.5,0.1,0.1),(0.5,0.1,0.1))	((0.95,0.1,0),(0.05,0,0.1))
A_2	((0.1,0.05,0.1),(0.9,0.1,0.05))	((0.9,0.05,0.1),(0.1,0.05,0.05))	((0.2,0.1,0.05),(0.8,0.05,0.1))	((0.5,0.1,0.1),(0.5,0.1,0.1))	((0.8,0.1,0.05),(0.2,0.05,0.1))
A_3	((0.05,0,0.1),(0.95,0.05,0))	((0.5,0.1,0.1),(0.5,0.1,0.1))	((0.2,0.1,0.05),(0.8,0.05,0.1))	((0.2,0.1,0.05),(0.8,0.05,0.1))	((0.9,0.05,0.1),(0.1,0.05,0.05))
A_4	((0.2,0.1,0.05),(0.8,0.05,0.1))	((0.5,0.1,0.1),(0.5,0.1,0.1))	((0.5,0.1,0.1),(0.5,0.1,0.1))	((0.2,0.1,0.05),(0.8,0.05,0.1))	((0.95,0.1,0),(0.05,0,0.1))
A_5	((0.5,0.1,0.1),(0.5,0.1,0.1))	((0.2,0.1,0.05),(0.8,0.05,0.1))	((0.5,0.1,0.1),(0.5,0.1,0.1))	((0.5,0.1,0.1),(0.5,0.1,0.1))	((0.8,0.1,0.05),(0.2,0.05,0.1))
A_6	((0.3,0.1,0.05),(0.7,0.05,0.1))	((0.5,0.1,0.1),(0.5,0.1,0.1))	((0.2,0.1,0.05),(0.8,0.05,0.1))	((0.2,0.1,0.05),(0.8,0.05,0.1))	((0.9,0.05,0.1),(0.1,0.05,0.05))
A_7	((0.7,0.05,0.1),(0.3,0.1,0.05))	((0.2,0.1,0.05),(0.8,0.05,0.1))	((0.2,0.1,0.05),(0.8,0.05,0.1))	((0.7,0.05,0.1),(0.3,0.1,0.05))	((0.8,0.1,0.05),(0.2,0.05,0.1))
A_8	((0.8,0.1,0.05),(0.2,0.05,0.1))	((0.2,0.1,0.05),(0.8,0.05,0.1))	((0.8,0.1,0.05),(0.2,0.05,0.1))	((0.8,0.1,0.05),(0.2,0.05,0.1))	((0.7,0.05,0.1),(0.3,0.1,0.05))
A_9	((0.7,0.05,0.1),(0.3,0.1,0.05))	((0.9,0.05,0.1),(0.1,0.05,0.05))	((0.5,0.1,0.1),(0.5,0.1,0.1))	((0.8,0.1,0.05),(0.2,0.05,0.1))	((0.8,0.1,0.05),(0.2,0.05,0.1))
A_{10}	((0.8,0.1,0.05),(0.2,0.05,0.1))	((0.2,0.1,0.05),(0.8,0.05,0.1))	((0.5,0.1,0.1),(0.5,0.1,0.1))	((0.8,0.1,0.05),(0.2,0.05,0.1))	((0.7,0.05,0.1),(0.3,0.1,0.05))
A_{11}	((0.9,0.05,0.1),(0.1,0.05,0.05))	((0.05,0,0.1),(0.95,0.05,0))	((0.5,0.1,0.1),(0.5,0.1,0.1))	((0.9,0.05,0.1),(0.1,0.05,0.05))	((0.3,0.1,0.05),(0.7,0.05,0.1))
A_{12}	((0.9,0.05,0.1),(0.1,0.05,0.05))	((0.05,0,0.1),(0.95,0.05,0))	((0.7,0.05,0.1),(0.3,0.1,0.05))	((0.9,0.05,0.1),(0.1,0.05,0.05))	((0.3,0.1,0.05),(0.7,0.05,0.1))
A_{13}	((0.95,0.1,0),(0.05,0,0.1))	((0.2,0.1,0.05),(0.8,0.05,0.1))	((0.8,0.1,0.05),(0.2,0.05,0.1))	((0.95,0.1,0),(0.05,0,0.1))	((0.7,0.05,0.1),(0.3,0.1,0.05))
A_{14}	((0.8,0.1,0.05),(0.2,0.05,0.1))	((0.2,0.1,0.05),(0.8,0.05,0.1))	((0.8,0.1,0.05),(0.2,0.05,0.1))	((0.8,0.1,0.05),(0.2,0.05,0.1))	((0.3,0.1,0.05),(0.7,0.05,0.1))

	C_6	C_7	C_8	C_9	C_{10}
A_1	((0.2,0.1,0.05),(0.8,0.05,0.1))	((0.5,0.1,0.1),(0.5,0.1,0.1))	((0.2,0.1,0.05),(0.8,0.05,0.1))	((0.8,0.1,0.05),(0.2,0.05,0.1))	((0.2,0.1,0.05),(0.8,0.05,0.1))
A_2	((0.2,0.1,0.05),(0.8,0.05,0.1))	((0.2,0.1,0.05),(0.8,0.05,0.1))	((0.8,0.1,0.05),(0.2,0.05,0.1))	((0.95,0.1,0),(0.05,0,0.1))	((0.7,0.05,0.1),(0.3,0.1,0.05))
A_3	((0.05,0,0.1),(0.95,0.05,0))	((0.2,0.1,0.05),(0.8,0.05,0.1))	((0.95,0.1,0),(0.05,0,0.1))	((0.95,0.1,0),(0.05,0,0.1))	((0.1,0.05,0.1),(0.9,0.1,0.05))
A_4	((0.05,0,0.1),(0.95,0.05,0))	((0.3,0.1,0.05),(0.7,0.05,0.1))	((0.95,0.1,0),(0.05,0,0.1))	((0.9,0.05,0.1),(0.1,0.05,0.05))	((0.1,0.05,0.1),(0.9,0.1,0.05))
A_5	((0.5,0.1,0.1),(0.5,0.1,0.1))	((0.5,0.1,0.1),(0.5,0.1,0.1))	((0.5,0.1,0.1),(0.5,0.1,0.1))	((0.5,0.1,0.1),(0.5,0.1,0.1))	((0.5,0.1,0.1),(0.5,0.1,0.1))
A_6	((0.3,0.1,0.05),(0.7,0.05,0.1))	((0.3,0.1,0.05),(0.7,0.05,0.1))	((0.7,0.05,0.1),(0.3,0.1,0.05))	((0.9,0.05,0.1),(0.1,0.05,0.05))	((0.1,0.05,0.1),(0.9,0.1,0.05))
A_7	((0.5,0.1,0.1),(0.5,0.1,0.1))	((0.8,0.1,0.05),(0.2,0.05,0.1))	((0.2,0.1,0.05),(0.8,0.05,0.1))	((0.7,0.05,0.1),(0.3,0.1,0.05))	((0.8,0.1,0.05),(0.2,0.05,0.1))
A_8	((0.7,0.05,0.1),(0.3,0.1,0.05))	((0.7,0.05,0.1),(0.3,0.1,0.05))	((0.3,0.1,0.05),(0.7,0.05,0.1))	((0.7,0.05,0.1),(0.3,0.1,0.05))	((0.9,0.05,0.1),(0.1,0.05,0.05))
A_9	((0.7,0.05,0.1),(0.3,0.1,0.05))	((0.3,0.1,0.05),(0.7,0.05,0.1))	((0.9,0.05,0.1),(0.1,0.05,0.05))	((0.8,0.1,0.05),(0.2,0.05,0.1))	((0.5,0.1,0.1),(0.5,0.1,0.1))
A_{10}	((0.8,0.1,0.05),(0.2,0.05,0.1))	((0.8,0.1,0.05),(0.2,0.05,0.1))	((0.2,0.1,0.05),(0.8,0.05,0.1))	((0.8,0.1,0.05),(0.2,0.05,0.1))	((0.7,0.05,0.1),(0.3,0.1,0.05))
A_{11}	((0.9,0.05,0.1),(0.1,0.05,0.05))	((0.9,0.05,0.1),(0.1,0.05,0.05))	((0.05,0,0.1),(0.95,0.05,0))	((0.7,0.05,0.1),(0.3,0.1,0.05))	((0.9,0.05,0.1),(0.1,0.05,0.05))
A_{12}	((0.95,0.1,0),(0.05,0,0.1))	((0.9,0.05,0.1),(0.1,0.05,0.05))	((0.2,0.1,0.05),(0.8,0.05,0.1))	((0.7,0.05,0.1),(0.3,0.1,0.05))	((0.9,0.05,0.1),(0.1,0.05,0.05))
A_{13}	((0.95,0.1,0),(0.05,0,0.1))	((0.9,0.05,0.1),(0.1,0.05,0.05))	((0.3,0.1,0.05),(0.7,0.05,0.1))	((0.8,0.1,0.05),(0.2,0.05,0.1))	((0.8,0.1,0.05),(0.2,0.05,0.1))
A_{14}	((0.9,0.05,0.1),(0.1,0.05,0.05))	((0.8,0.1,0.05),(0.2,0.05,0.1))	((0.2,0.1,0.05),(0.8,0.05,0.1))	((0.7,0.05,0.1),(0.3,0.1,0.05))	((0.9,0.05,0.1),(0.1,0.05,0.05))

Table A5: Criteria weights for sensitivity analysis given for each decision-matrix

	Case 1	C_1	C_2	C_3	C_4	C_5	C_6	C_7	C_8	C_9	C_{10}
Decision matrix 1	1	0.0213	0.1854	0.0927	0.0032	0.2571	0.1229	0.0894	0.1182	0.0398	0.0700
	2	0.1025	0.2043	0.0104	0.0347	0.0115	0.1662	0.1241	0.0819	0.1028	0.1616
	3	0.2301	0.1009	0.1193	0.1012	0.0278	0.0740	0.0835	0.1263	0.0901	0.0468
	4	0.0062	0.2314	0.0882	0.1425	0.0011	0.1209	0.0533	0.1467	0.1012	0.1085
	5	0.0771	0.1508	0.0083	0.2332	0.0549	0.0582	0.1195	0.0523	0.1017	0.1440
	6	0.1145	0.0332	0.2020	0.0827	0.1013	0.1235	0.0458	0.0612	0.1119	0.1239
	7	0.0603	0.0981	0.1519	0.1332	0.0145	0.1822	0.0735	0.0627	0.1016	0.1220
	8	0.0425	0.1308	0.1019	0.1821	0.0183	0.1097	0.0593	0.1402	0.0957	0.1195
	9	0.1203	0.0482	0.0654	0.2185	0.0053	0.1011	0.1483	0.0919	0.0668	0.1342
	10	0.0119	0.1295	0.2037	0.0164	0.0892	0.1285	0.0723	0.1221	0.1004	0.1260
Decision matrix 2	1	0.1891	0.0115	0.1054	0.0012	0.1138	0.1293	0.0424	0.1534	0.1019	0.1520
	2	0.0732	0.0815	0.0233	0.1295	0.1934	0.1021	0.0064	0.1427	0.1149	0.1330
	3	0.1205	0.1003	0.0714	0.0115	0.0826	0.2187	0.0391	0.0862	0.0780	0.1917
	4	0.0302	0.1231	0.1895	0.0771	0.0924	0.0081	0.1019	0.1255	0.0942	0.1580
	5	0.0410	0.0029	0.1315	0.0733	0.1337	0.0576	0.1402	0.0641	0.1067	0.2490
	6	0.0143	0.1265	0.1321	0.0887	0.0752	0.1126	0.0679	0.1205	0.0634	0.1988
	7	0.1574	0.0456	0.1287	0.0125	0.0953	0.1329	0.0612	0.1048	0.0885	0.1731
	8	0.0211	0.1379	0.0423	0.1326	0.1107	0.1211	0.0842	0.0515	0.1539	0.1447
	9	0.1062	0.1710	0.0294	0.0193	0.1187	0.0534	0.0941	0.1232	0.1018	0.1829
	10	0.1235	0.0214	0.0917	0.1520	0.0892	0.1123	0.0775	0.1245	0.0489	0.1590
Decision matrix 3	1	0.0117	0.0735	0.1424	0.1281	0.1922	0.0059	0.0965	0.0934	0.1043	0.1520
	2	0.1003	0.1491	0.0115	0.0728	0.1052	0.1391	0.0193	0.1482	0.0843	0.1702
	3	0.0872	0.1123	0.0619	0.1732	0.0221	0.1015	0.1350	0.0911	0.1087	0.1070
	4	0.0105	0.1702	0.1194	0.0676	0.1032	0.0839	0.1125	0.1401	0.0916	0.1010
	5	0.0503	0.0809	0.1572	0.0291	0.1204	0.1235	0.0762	0.0893	0.0957	0.1774
	6	0.1342	0.0741	0.1018	0.0453	0.1387	0.1035	0.0619	0.0772	0.1050	0.1583
	7	0.1159	0.0071	0.1337	0.1452	0.0903	0.1221	0.0659	0.0893	0.0812	0.1493
	8	0.0245	0.1648	0.0742	0.1049	0.1192	0.1378	0.0607	0.1093	0.0481	0.1565
	9	0.0871	0.0569	0.1425	0.0958	0.0304	0.1236	0.1069	0.1212	0.0987	0.1369
	10	0.0037	0.0910	0.1324	0.1567	0.0431	0.1049	0.0738	0.1231	0.1170	0.1543
Decision matrix 4	1	0.0214	0.1223	0.1437	0.1091	0.0124	0.1825	0.0951	0.0716	0.1072	0.1347
	2	0.0941	0.0723	0.1382	0.1625	0.0916	0.0053	0.1157	0.1018	0.0811	0.1374
	3	0.1502	0.0617	0.1243	0.0929	0.0121	0.1823	0.0815	0.0906	0.0692	0.1352
	4	0.0285	0.1418	0.0087	0.1269	0.0823	0.1235	0.1047	0.1101	0.0892	0.1843
	5	0.0171	0.0962	0.1367	0.0925	0.1531	0.0312	0.0873	0.1042	0.0856	0.1961
	6	0.1212	0.0531	0.0775	0.1692	0.1023	0.0754	0.1149	0.0812	0.0917	0.1135
	7	0.0663	0.1875	0.0932	0.0157	0.1403	0.0548	0.1115	0.0927	0.0418	0.1962
	8	0.1014	0.1032	0.0215	0.1327	0.1342	0.0498	0.0723	0.1231	0.0748	0.1870
	9	0.0547	0.1381	0.1523	0.0182	0.0716	0.1572	0.0369	0.1073	0.0818	0.1819
	10	0.1310	0.1192	0.0875	0.1261	0.0202	0.0741	0.1083	0.0816	0.1034	0.1486

Table A6: Expert weights for sensitivity analysis

Case	E_1	E_2	E_3	E_4
1	0.12	0.34	0.18	0.36
2	0.45	0.05	0.25	0.25
3	0.10	0.20	0.30	0.40
4	0.60	0.10	0.20	0.10
5	0.33	0.17	0.25	0.25
6	0.05	0.15	0.50	0.30
7	0.27	0.13	0.40	0.20
8	0.08	0.22	0.18	0.52
9	0.14	0.36	0.10	0.40
10	0.50	0.20	0.15	0.15

Table A7: Sensitivity analysis with respect to criteria weights

	Case 1		Case 2		Case 3		Case 4		Case 5		Case 6		Case 7		Case 8		Case 9		Case 10	
Alt	Ideal	Rank	Ideal	Rank	Ideal	Rank	Ideal	Rank	Ideal	Rank	Ideal	Rank	Ideal	Rank	Ideal	Rank	Ideal	Rank	Ideal	Rank
A_1	0.3216	14	0.3782	13	0.3811	14	0.2870	13	0.4801	11	0.3688	14	0.5648	11	0.3871	13	0.6026	13	0.5127	13
A_2	0.8660	2	1.0000	1	0.9657	4	1.0000	1	0.7673	4	0.6638	8	0.8026	6	1.0000	1	0.9195	5	0.9292	2
A_3	0.8078	5	0.7708	5	0.8286	7	0.5900	8	0.7337	6	0.7583	4	0.7341	7	0.8212	6	0.8393	7	0.8899	5
A_4	1.0000	1	0.9281	2	1.0000	1	0.6750	6	1.0000	1	1.0000	1	1.0000	1	0.9887	2	0.9897	3	1.0000	1
A_5	0.3393	13	0.3306	14	0.4890	13	0.2047	14	0.3773	14	0.3864	13	0.4485	14	0.3278	14	0.5141	14	0.4410	14
A_6	0.6682	9	0.6301	9	0.7579	9	0.4402	10	0.7893	3	0.7856	2	0.7134	8	0.6867	9	0.7699	9	0.7733	10
A_7	0.5611	11	0.4971	12	0.5191	12	0.3845	12	0.4694	12	0.5650	11	0.5415	13	0.5347	11	0.6088	12	0.6531	11
A_8	0.6203	10	0.6096	10	0.7677	8	0.4853	9	0.4973	10	0.6055	9	0.6326	10	0.6388	10	0.7880	8	0.7797	9
A_9	0.8403	3	0.7518	6	0.7123	10	0.7145	3	0.6539	9	0.5886	10	0.8110	5	0.7175	8	0.7422	10	0.9025	4
A_{10}	0.5254	12	0.5047	11	0.6405	11	0.4176	11	0.4001	13	0.5494	12	0.5610	12	0.5285	12	0.6613	11	0.6435	12
A11	0.8103	4	0.7430	7	0.8622	5	0.7360	2	0.7368	5	0.7524	5	0.9435	2	0.9034	3	0.9919	2	0.9115	3
A12	0.8013	6	0.7178	8	0.8444	6	0.7114	4	0.7267	7	0.7414	6	0.9243	3	0.8407	4	1.0000	1	0.8567	8
A13	0.7547	8	0.8053	4	0.9762	2	0.6675	7	0.6558	8	0.7210	7	0.7087	9	0.7926	7	0.8745	6	0.8749	6
A14	0.7975	7	0.8422	3	0.9711	3	0.6928	5	0.8387	2	0.7708	3	0.8326	4	0.8367	5	0.9888	4	0.8599	7

Table A8: Sensitivity analysis with respect to expert weights

	Case 1		Case 2		Case 3		Case 4		Case 5		Case 6		Case 7		Case 8		Case 9		Case 10	
Alt	Ideal	Rank	Ideal	Rank	Ideal	Rank	Ideal	Rank	Ideal	Rank	Ideal	Rank	Ideal	Rank	Ideal	Rank	Ideal	Rank	Ideal	Rank
A1	0.0442	14	0.0529	14	0.0485	14	0.0514	14	0.0494	14	0.0500	14	0.0506	14	0.0479	14	0.0436	14	0.0485	14
A2	0.7100	3	0.7157	3	0.7128	3	0.7147	3	0.7134	3	0.7137	3	0.7141	3	0.7124	3	0.7096	3	0.7128	3
A3	0.4632	7	0.4624	7	0.4629	7	0.4626	7	0.4628	7	0.4628	7	0.4627	7	0.4630	7	0.4632	7	0.4629	7
A4	0.2938	13	0.2988	13	0.2963	13	0.2980	13	0.2968	13	0.2971	13	0.2975	13	0.2959	13	0.2935	13	0.2963	13
A5	0.5323	6	0.5225	6	0.5275	6	0.5242	6	0.5265	6	0.5258	6	0.5252	6	0.5282	6	0.5330	6	0.5275	6
A6	0.3061	12	0.3028	12	0.3045	12	0.3034	12	0.3041	12	0.3039	12	0.3037	12	0.3047	12	0.3064	12	0.3045	12
A7	0.3949	9	0.3899	9	0.3925	9	0.3907	9	0.3919	9	0.3916	9	0.3912	9	0.3928	9	0.3953	9	0.3925	9
A8	0.4372	8	0.4364	8	0.4368	8	0.4365	8	0.4367	8	0.4366	8	0.4366	8	0.4368	8	0.4372	8	0.4368	8
A9	0.5448	4	0.5392	4	0.5420	4	0.5401	4	0.5415	4	0.5411	4	0.5407	4	0.5424	4	0.5452	4	0.5420	4
A10	0.5342	5	0.5301	5	0.5322	5	0.5308	5	0.5318	5	0.5315	5	0.5312	5	0.5325	5	0.5345	5	0.5322	5
A11	0.9224	2	0.9217	2	0.9221	2	0.9218	2	0.9220	2	0.9219	2	0.9219	2	0.9221	2	0.9224	2	0.9221	2
A12	1.0000	1	1.0000	1	1.0000	1	1.0000	1	1.0000	1	1.0000	1	1.0000	1	1.0000	1	1.0000	1	1.0000	1
A13	0.3549	11	0.3527	11	0.3538	11	0.3531	11	0.3536	11	0.3535	11	0.3533	11	0.3540	11	0.3550	11	0.3538	11
A14	0.3636	10	0.3599	10	0.3617	10	0.3605	10	0.3614	10	0.3611	10	0.3609	10	0.3620	10	0.3638	10	0.3617	10
